# Quantitative Predictions of Binding Free Energy Changes in Drug-Resistant Influenza Neuraminidase

**DOI:** 10.1371/journal.pcbi.1002665

**Published:** 2012-08-30

**Authors:** Daniel R. Ripoll, Ilja V. Khavrutskii, Sidhartha Chaudhury, Jin Liu, Robert A. Kuschner, Anders Wallqvist, Jaques Reifman

**Affiliations:** 1 Department of Defense Biotechnology High Performance Computing Software Applications Institute, Telemedicine and Advanced Technology Research Center, US Army Medical Research and Materiel Command, Fort Detrick, Frederick, Maryland, United States of America; 2 Walter Reed Army Institute of Research, Emerging Infectious Diseases Research Unit, Silver Spring, Maryland, United States of America; University of Maryland, United States of America

## Abstract

Quantitatively predicting changes in drug sensitivity associated with residue mutations is a major challenge in structural biology. By expanding the limits of free energy calculations, we successfully identified mutations in influenza neuraminidase (NA) that confer drug resistance to two antiviral drugs, zanamivir and oseltamivir. We augmented molecular dynamics (MD) with Hamiltonian Replica Exchange and calculated binding free energy changes for H274Y, N294S, and Y252H mutants. Based on experimental data, our calculations achieved high accuracy and precision compared with results from established computational methods. Analysis of 15 µs of aggregated MD trajectories provided insights into the molecular mechanisms underlying drug resistance that are at odds with current interpretations of the crystallographic data. Contrary to the notion that resistance is caused by mutant-induced changes in hydrophobicity of the binding pocket, our simulations showed that drug resistance mutations in NA led to subtle rearrangements in the protein structure and its dynamics that together alter the active-site electrostatic environment and modulate inhibitor binding. Importantly, different mutations confer resistance through different conformational changes, suggesting that a generalized mechanism for NA drug resistance is unlikely.

## Author Summary

The capacity of the influenza virus to rapidly mutate and render resistance to a handful of FDA approved neuraminidase (NA) inhibitors represents a significant human health concern. To gain an atomic-level understanding of the mechanisms behind drug resistance, we applied a novel computational approach to characterize resistant NA mutations. These results are comparable in accuracy and precision with the best experimental measurements presently available. To the best of our knowledge, this is the first time that a rigorous computational method has attained the level of certainty needed to predict subtle changes in binding free energies conferred by mutations. Analysis of our simulation data provided a thorough description of the thermodynamics of the binding process for different NA-inhibitor complexes, with findings that in some cases challenge current views based on interpretations of the crystallographic data. While we did not find a generalized mechanism of NA resistance, we identified key differences between oseltamivir and zanamivir that discriminate their responses to the three mutations we considered, namely H274Y, N294S and Y252H. It is worth noting that our approach can be broadly applied to predict resistant mutations to existing and newly developed drugs in other important drug targets.

## Introduction

Current plans for managing future influenza pandemics include the use of therapeutic and prophylactic drugs, such as zanamivir [1] and oseltamivir [2], that target the virus surface glycoprotein neuraminidase (NA) [3]. Inhibition of NA reduces the spread of the virus in the respiratory tract by interfering with the release of progeny virions from infected host cells. A handful of drug-resistant strains have recently emerged due to antigenic drift [4], [5], [6]. NA in these strains contains a series of mutations that do not significantly alter its function, yet render it resistant to inhibition. These mutations lead to a small (1–3 kcal/mol) decrease in the high-affinity binding of these inhibitors that is sufficient to restore *in vivo* viral propagation. Understanding how different NA mutations confer drug resistance is a critical step in discovering new drugs to safeguard against future influenza pandemics.

NAs from different influenza subtypes exhibit a variety of resistance mutations and these mutations can affect inhibitors differently. For example, the R292K mutation in N2 NAs confers resistance to oseltamivir [7], but in highly similar N1 NAs such mutation remains drug sensitive [8]. These and other complex patterns of resistance can only be explained by the interactions between the binding site and the inhibitors. Previous biochemical [9] and structural studies [10] have implicated the rearrangement of certain binding-site residues as the mechanism of drug resistance in NA. For example, bulky substitutions at H274 result in a conformational shift of the neighboring E276, which alters a hydrophobic pocket that specifically disrupts oseltamivir binding. While such structure-based explanations are plausible, a critical evaluation of these hypotheses requires atomic-scale models that accurately reflect the microscopic structural mechanisms guiding NA-inhibitor interactions.

X-ray crystallography provides high-resolution structures of NA-inhibitor complexes. Although such structures are vital to our understanding of NA-inhibitor interactions, the atomic coordinates themselves lend little direct insight into the underlying thermodynamics of drug resistance. There are numerous examples of crystal structures of proteins with drug resistance mutations, such as of HIV-1 protease [11], that show only minor structural differences when compared to the drug-sensitive wild type (WT) structure and do not reveal any readily apparent mechanism of resistance. Numerous drug resistance mutations in NA fall outside of the immediate binding pocket, and structures of the drug-resistant H274Y and N294S mutants co-crystallized with oseltamivir and zanamivir reveal binding-site conformations that are virtually identical to WT [10]. Molecular simulations that rigorously model the microscopic structure and thermodynamics [12], [13], [14] of NA-inhibitor interactions may provide insight into the mechanisms of drug resistance that elude traditional structure-based approaches.

Accurately modeling the thermodynamic consequences of mutations that alter protein function, such as in drug resistance, is a major challenge in structural biology. The change in binding free energy associated with a drug resistance mutation is a result of systemic shifts across the totality of structural conformations that impact which biochemical interactions are accessible in the wild-type and the mutant protein systems. Due to the staggering conformational complexity of a protein-inhibitor complex, direct and exhaustive modeling of this entire system is computationally unfeasible. To overcome such difficulties, two types of approaches for predicting free-energy changes from point mutations have been developed: empirical approaches, which apply highly trained score functions that approximate the free energy of a given structure, and simulation-based approaches, which combine extensive stochastic sampling with statistical mechanics-based calculations to estimate free energies. These approaches have been reviewed extensively elsewhere [15], [16], [17].

While empirical approaches have been moderately successful at identifying mutations along interfacial residues that disrupt binding, they fail to identify the numerous mutations outside of the interface where the effects are presumably smaller [18]. Even the most rigorous simulation-based methods currently available, such as Thermodynamic Integration (TI) and the closely related Free Energy Perturbation (FEP) [12], [13], [19], [20], [21], [22], may lack the accuracy and precision to assess small changes to otherwise large binding free energies. These methods, which, in theory, should capture the thermodynamic effects of protein mutations, have been applied to compute absolute binding free energies of several small molecules to wild type and mutant enzymes, including T4 lysozyme and NA [23], [24], [25], [26]. However, straightforward applications of these techniques to large, complex systems are hampered by significant sampling issues. These issues are particularly severe in systems with hindered conformational transitions associated with ligand binding, which often render the resulting absolute binding free energy calculations unreliable [27], [28], [29], [30]. Conventional methods for calculating relative binding free energies across a series of related compounds avoid many of the sampling issues associated with absolute binding free energy calculations [31], however, they are typically not directly applicable to assessing the effects of mutations on binding of the same compound.

Successful modeling of the thermodynamics of large, complex systems, such as NA, requires careful selection of both the conformational sampling strategy and the appropriate reference states in order to obtain precise and accurate estimates of free energy changes. We recently described a novel implementation of the Hamiltonian Replica Exchange (HREX) molecular dynamics (MD) method [31] that uses an alchemical thermodynamic pathway to arrive at reliable free energy calculations. Here, we adapted this approach to incorporate residue mutations into the thermodynamic cycle. Instead of estimating changes of binding free energies of different compounds with respect to the same protein, we estimated free energy changes for mutating a residue in the bound and unbound wild type protein. We applied this method to several such pathways to predict the binding free energy changes (ΔΔ*G*) of a set of mutations in H5N1 NA that have been experimentally tested for drug resistance. We successfully identified drug resistance mutations in NA using a judiciously chosen thermodynamic path within the HREX framework.

For this work, we adapted the criterion introduced by Kortemme et al. [32] to classify a mutation as drug resistant when its calculated ΔΔ*G* exceeded +1 kcal/mol. Based on this criterion, the experimentally observed NA mutations N294S, H274Y, and Y252H reveal different resistance patterns with respect to oseltamivir and zanamivir [10]. We explored the capabilities of our approach and alternate ones, including those from previously published work [33], [34], to produce accurate and precise ΔΔ*G* estimates consistent with the experimental data [10].

Analysis of over 15 µs of aggregate MD simulation data revealed that different mutations confer resistance through different conformational changes in the active site. Unexpectedly, we found no evidence supporting the previously reported role of hydrophobic interactions with the oseltamivir tail [10]. Instead, we hypothesize that drug resistance arises from rearrangements of several charged residues that alter the electrostatic environment within the binding site and disrupt inhibitor binding. The complexity of the observed structural perturbations highlights the importance of atomic-level structural details and suggests that identification of a generalized theory of resistance is unlikely.

## Results/Discussion

### Binding free energy changes

We computed *relative* instead of *absolute* binding free energy changes using Single Reference Thermodynamic Integration (SRTI) [31]. Computing relative ΔΔ*G*s requires measuring the free energy change along an alchemical thermodynamic path linking the WT to the mutant protein for the ligand-bound and ligand-free states independently, which requires only a partial ‘decoupling’ of the mutating residues and/or ligand along that alchemical path. In contrast, absolute ΔΔ*G* computations entail measuring the free energy change along an alchemical thermodynamic path connecting the ligand-bound and ligand-free states, which requires a complete decoupling of the ligand from the protein [35]. Previous MD simulations of NA [36], [37] revealed substantial binding-induced conformational changes along a 150-residue loop. A complete decoupling of the ligand [25] would necessitate extensive sampling of this large conformational transition, making reliable free energy predictions practically impossible. By avoiding the need to explicitly model this binding-induced conformational change, the relative SRTI approach is better suited for ΔΔ*G* calculations for NA.

#### ΔΔ*G* calculations using SRTI

**Figure 1:**
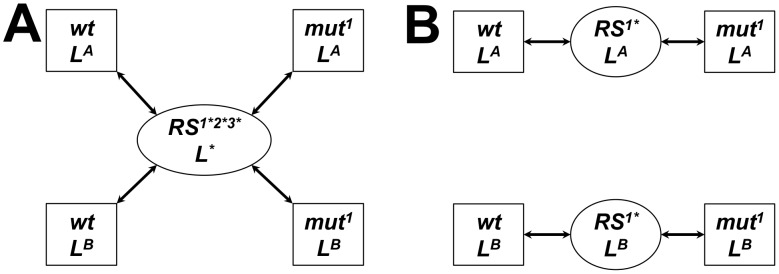
Alchemical thermodynamic paths using SRMM (A) and SRSM (B) in the bound state between wild type (*wt*) and a mutant (*mut^1^*). The paths (arrows) between end states (squares) going through nonphysical reference states (ovals) are shown. The SRMM uses a common reference state ‘hub’ for all mutations and ligands (RS^1*2*3*^ L^*^); SRSM uses a mutation and ligand-specific reference state (RS^1*^ L^X^). Decoupled residues and ligands are noted by an ‘*’. Thermodynamic paths in the unbound state have a similar form but without the ligand.

We estimated differences in the binding free energies of two ligands with four NA proteins (three mutants and one WT). To calculate ΔΔ*G*s, we constructed a set of alchemical thermodynamic paths that pass through a common unphysical reference state (RS) shared by all four proteins and both ligands (Fig. 1A). This RS ‘hub’ allowed us to thermodynamically link the binding free energy changes for all the protein/ligand combinations simultaneously, yielding the least computationally expensive set of simulations. In order to minimize perturbations along the alchemical paths, we constructed a RS that resulted in decoupling of only the regions of the mutating residues not shared by both residue types and regions of the ligand not shared by both inhibitors. This was done by replacing the mutating residues with unphysical “pseudo” residues in the RS protein and replacing the inhibitor with an unphysical pseudo-ligand derived from the shared inhibitor scaffold in the RS ligand (further details provided in Supporting Information [SI] Section 1*e*). We refer to these calculations as the single-reference multiple mutants (SRMM) approach. Table 1 summarizes the computed ΔΔ*G*s relative to the WT for each inhibitor using SRMM with standard MD. For all drug/mutant combinations, the results showed low accuracy and low precision with an overall root mean squared (RMS) error and RMS standard deviation of 4.2 kcal/mol and 7.4 kcal/mol, respectively, and failed to reproduce experimental observations with any certainty. Structural analysis of these simulations showed that the large standard deviations resulted from significant perturbations to the ligand pose that were mainly due to the decoupling of the flexible tail of the ligand in the RS.

**Table 1:** Comparison of experimental ΔΔ*G* in oseltamivir and zanamivir for three NA mutations with estimates obtained using different computational approaches.

Method	H274Y		N294S		Y252H		RMSE
	ΔΔ*G*, kcal/mol		ΔΔ*G*, kcal/mol		ΔΔ*G*, kcal/mol		(RMSD), kcal/mol
	zanamivir	oseltamivir	zanamivir	oseltamivir	zanamivir	oseltamivir	
Experimental^a^	**0.4 (0.1)**	**3.3 (0.2)** *	**1.2 (0.1)** *	**2.6 (0.2)** *	**0.1 (0.2)**	**−1.4 (0.1)**	N/A (0.2)
SRMM	−5.8 (7.4)	0.7 (7.0)	8.2 (7.7)	5.8 (6.2)	−0.1 (8.7)	−0.9 (7.4)	4.2 (7.4)
SRSM	1.7 (2.9)	1.2 (3.0)	0.6 (2.0)	1.7 (1.9)	1.5 (1.7)	0.5 (1.5)	1.5 (2.2)
SRSM/HREX	1.3 (0.8)	4.1 (2.4)	2.3 (0.4)	2.2 (0.9)	0.6 (0.8)	0.7 (1.4)	1.1 (1.1)
MM-GBSA	6.2 (8.1)	0.9 (3.8)	5.7 (6.1)	−5.9 (3.6)	2.1 (2.9)	−1.9 (3.0)	4.8 (4.6)
MM-PBSA	8.4 (10.1)	3.0 (3.9)	5.8 (4.5)	−4.7 (3.2)	2.8 (3.1)	0.2 (2.6)	5.0 (4.6)
Rosetta	−0.4 (0.5)	0.8 (0.4)	−0.4 (0.3)	0.3 (0.2)	−0.1 (0.4)	0.0 (0.0)	1.7 (0.3)

a Values were derived from the data reported by Collins et al [10].

Standard deviations are shown in parentheses. Root mean squared error (RMSE) and the RMS Standard Deviation (RMSD) are provided.

‘*’ indicates experimentally determined drug resistant mutation. ‘N/A’ stands for *not applicable*.

While the SRMM approach minimized the number of simulations needed to calculate ΔΔ*G*s, the relatively high degree of decoupling associated with a single common RS undermined its accuracy. To reduce the uncertainty in ΔΔ*G* predictions, we constructed a set of alchemical thermodynamic paths that minimized the degree of decoupling in the unphysical reference states. This entailed constructing independent thermodynamic paths that connected mutant and WT proteins through reference states specific to each mutation and ligand (Fig. 1B). In these alchemical paths, only the single mutating residue was partially decoupled by using a reference state in which the mutating residue was represented by a pseudo-residue while all the other residues and the ligand remained fully physical. We refer to these simulations as the single reference single mutant (SRSM) approach. While this approach minimizes the extent of decoupling in the respective reference states, it effectively requires 50% more computational resources than the SRMM approach.

Table 1 lists the ΔΔ*G* estimates from the SRSM calculations using standard MD, which showed an overall RMS error and standard deviation of 1.5 kcal/mol and 2.2 kcal/mol, respectively. This represents a substantial improvement over the SRMM results. To test whether enhanced sampling with the SRSM approach would further improve the binding energy predictions, we augmented the SRSM calculations with HREX MD (SRSM/HREX). In five out of the six cases, the SRSM/HREX simulations correctly identified drug resistance mutations using our pre-defined criterion. Table 1 shows that this approach substantially improved the overall accuracy and precision of the predictions despite still being unable to capture the increased sensitivity of Y252H to oseltamivir.

The SRSM/HREX calculations reached a chemical accuracy of one kcal/mol and identified drug resistant mutants for both zanamivir and oseltamivir with high certainty. In agreement with experiments, SRSM/HREX predicted that Y252H shows no resistance to both inhibitors, N294S confers resistance to both inhibitors, and that H274Y confers resistance to oseltamivir. However, SRSM/HREX incorrectly classified H274Y as resistant to zanamivir. Overall, our binding free energy calculations constitute a clear advancement over previously published results using more approximate and less computationally intensive approaches [33], [34], [36].

#### Comparison with alternate methods for calculating ΔΔ*G*

In order to directly compare alternate methods with the more rigorous and computationally expensive SRTI, we calculated binding free energies changes using the Molecular Mechanics - Poisson Boltzmann Surface Area (MM-PBSA) and Generalized Born Surface Area (MM-GBSA) approaches [14], [38] (see *Materials and Methods*). Table 1 provides a summary of the resulting ΔΔ*G* calculations. Overall, the predictions from MM-PBSA/GBSA were significantly less accurate than those from the SRTI simulations, with an RMS error of 4.8 kcal/mol and 5.0 kcal/mol for MM-GBSA and MM-PBSA, respectively, and standard deviations of 4.6 kcal/mol in both cases. Previous studies using MM-GBSA, MM-PBSA, or Linear Interaction Energy (LIE) methods for oseltamivir and zanamivir binding to H274Y and N294S mutants have shown qualitative agreement with experimental data but with relatively low quantitative accuracy [33], [34], [39], [40].

The Rosetta procedure for estimating ΔΔ*G* (see *Materials and Methods*) was the least computationally expensive approach to predicting binding free energy changes. Our results show that although the Rosetta predictions had low standard deviations, it was unable to accurately predict the effect of any of the three mutations. Previous studies in a benchmark set of protein-protein interactions [41] have shown that the Rosetta approach is not well-suited to modeling mutations located beyond the immediate interface [18].

In summary, we present a series of SRTI simulations that gradually improved in accuracy and precision, with the SRSM/HREX simulations producing the best estimates of ΔΔ*G*s. This was a substantial improvement upon our initial SRMM approach and underscores the need for careful consideration in the choice of simulation techniques and thermodynamic paths in order to achieve the best results. In contrast, both MM-PBSA/GBSA and Rosetta failed to accurately predict the mutations that confer drug resistance. Ultimately, the goal of the MD simulations was to generate a thermodynamically accurate, atomic-scale model of NA-inhibitor interactions. Our derivation of ΔΔ*G*s using the SRSM/HREX simulations agreed well with experimental values, suggesting that we succeeded toward that end. Thus, we proceeded to carry out structural analyses of the composite simulation trajectories to identify the microscopic mechanisms underlying the observed free energy differences.

#### Identifying key NA-inhibitor interactions

**Figure 2:**
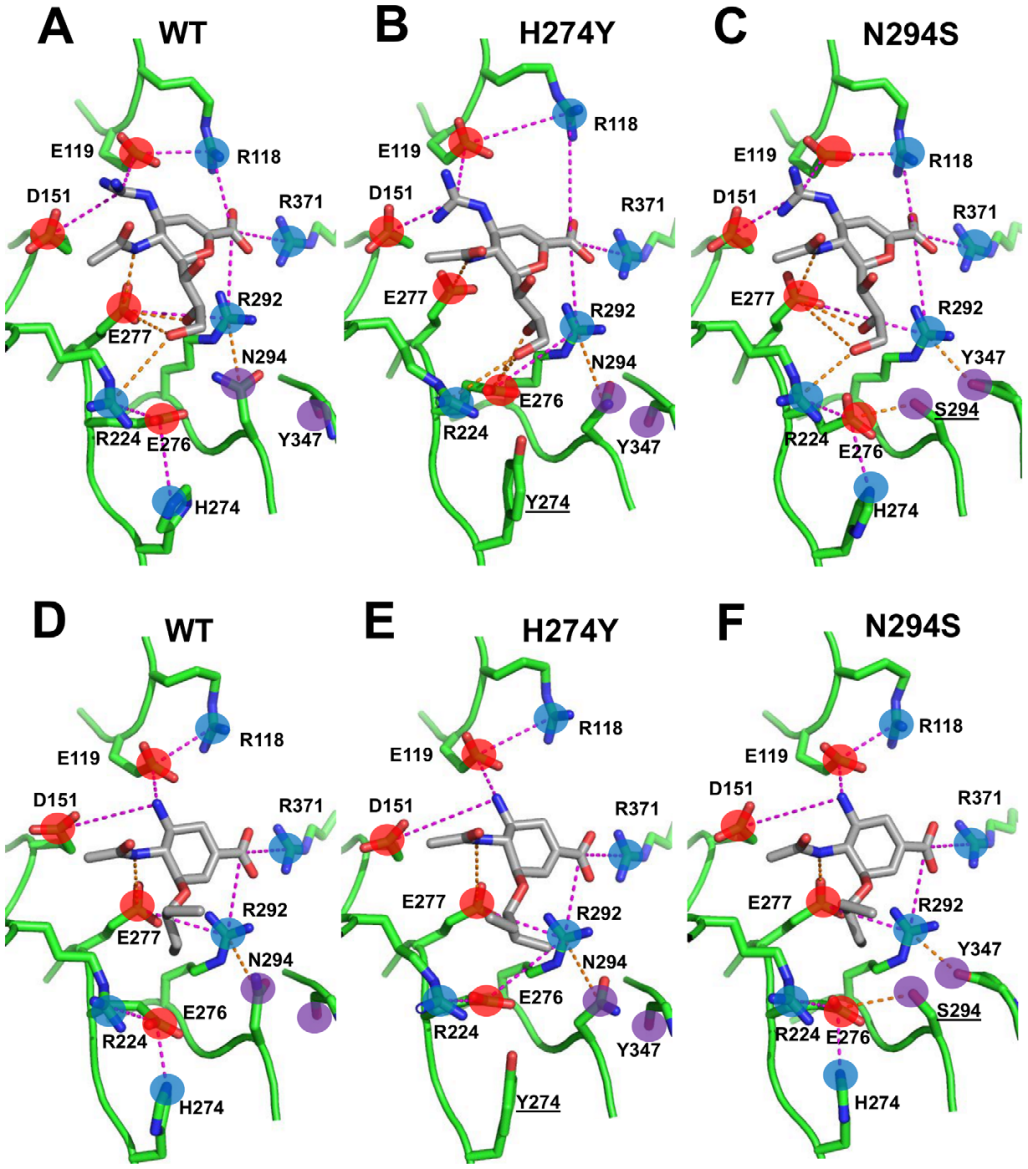
Representative structures for zanamivir (A, B and C) and oseltamivir (D, E and F) bound to WT and mutant NAs from the SRSM/HREX simulations. Salt-bridges and hydrogen bonds are depicted as magenta and orange dashed lines, respectively. Positively charged, negatively charged, and uncharged polar groups are noted as blue, red, and purple circles, respectively, and residues of interest are labeled. Mutated residues are underlined.

To identify which residues play a major role in NA-inhibitor interactions, we separately analyzed the composite WT trajectories of NA bound to zanamivir and oseltamivir. Since SRTI does not partition the computed free energy into the contribution from each residue, we used the average residue energies from the MM-GBSA calculations to quantify the energetic contribution of the residues in the binding pocket (Table S1). For residues that showed significant contribution to the binding energy, we calculated distance distributions for the inhibitor and neighboring residues to identify any systematic changes in biochemical interactions at the binding site. Further discussion is provided in SI Section 2. In addition, Fig. S3 in Text S1 shows an interaction map derived from these data for both zanamivir and oseltamivir and Fig. 2 shows representative structures for both inhibitor complexes.

### Molecular origins of drug resistance

Determining the molecular mechanisms of NA drug resistance involves identifying key protein structural features that underlie the thermodynamic differences in inhibitor binding observed in the simulation data. Such features may include changes in biochemical interactions in the NA-inhibitor complex, systematic shifts in the NA structure, and even subtle differences in the overall dynamics between WT and drug-resistant NA. A visual comparison between the crystal structures of NA in complex with zanamivir and oseltamivir revealed few apparent differences in NA-inhibitor interactions. Therefore, we analyzed the structural data derived from the SRSM/HREX simulations in order to identify reliable structural differences between WT and drug-resistant mutant trajectories.

Fig. 2 illustrates representative structures from the WT and drug-resistant mutant trajectories for zanamivir and oseltamivir, confirming the x-ray crystallography findings that the most prominent binding interactions are preserved. The negatively charged carboxyl group of both inhibitors maintained interactions with a basic triad formed by R118, R292, and R371. The positively charged ammonium and guanidinium groups of oseltamivir and zanamivir, respectively, maintained salt-bridges with the acidic E119, D151, and E227 residues (E227 is not displayed in Fig. 2 for purposes of clarity). Finally, the polar tail of zanamivir maintained some of the hydrogen bonds with R224, E276, and E277 in both WT and mutant forms. The long-range nature of these electrostatic interactions and the highly flexible nature of the binding site suggest that NA-inhibitor binding is highly sensitive to subtle, systematic rearrangements of the electrostatic environment caused by mutations beyond the immediate binding site. Our analysis identified several such rearrangements that may be critical to drug resistance.

#### The H274Y mutant

The SRSM/HREX simulations of the H274Y mutation yielded a ΔΔ*G* of binding for zanamivir and oseltamivir of +1.3 kcal/mol and +4.1 kcal/mol, respectively. This mutation replaces a positively charged histidine with a bulkier, uncharged tyrosine in the second residue layer around the binding site. We found that the H274Y mutation perturbed a number of intermolecular and intramolecular interactions within the binding pocket. These changes were largely localized to the charged residues E276, E277, and R224, which form the binding pocket for the inhibitor tail.

**Figure 3:**
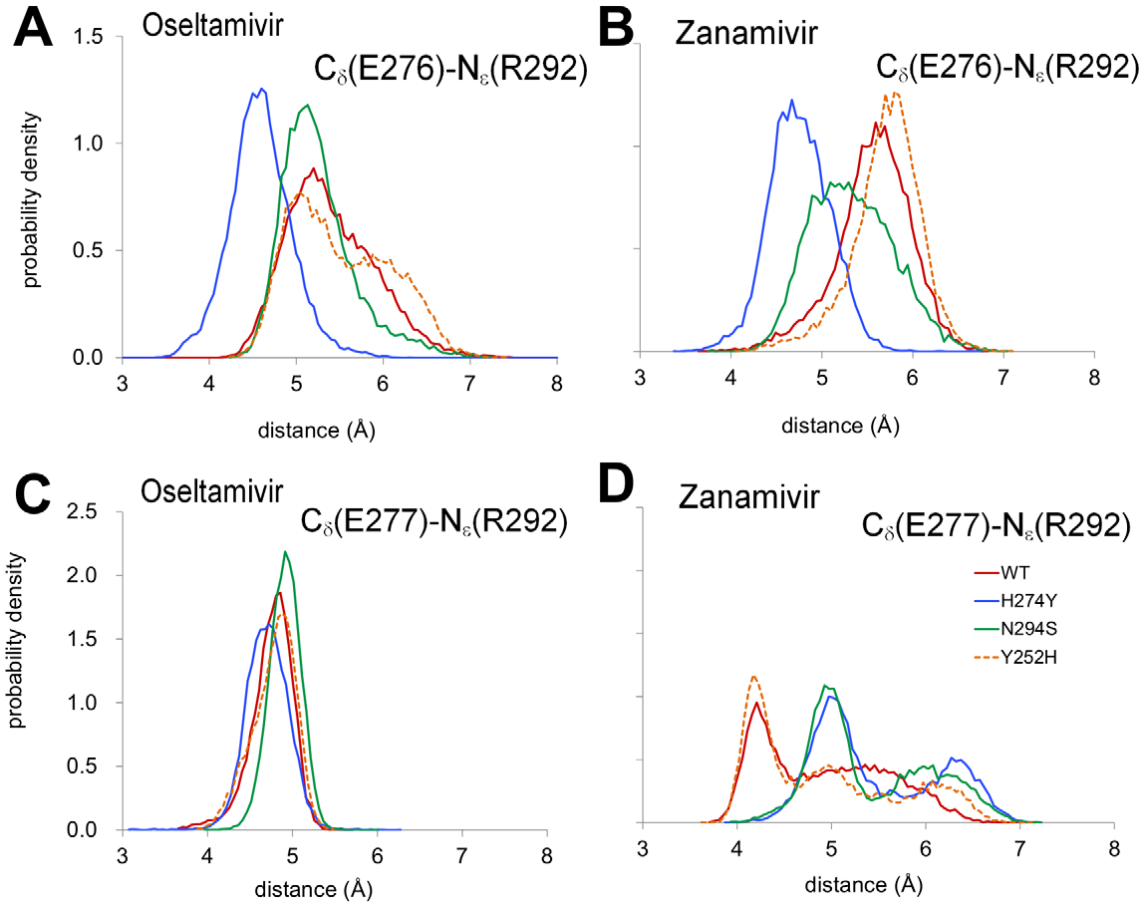
Comparison of WT and mutant dynamics. For WT and H274Y, N294S, and Y252H, distance distributions between the atoms C_δ_ of E276 and N_ε_ of R292 and between the atoms C_δ_ of E277 and N_ε_ of R292 are shown for oseltamivir (**A, C**) and zanamivir (**B, D**), respectively.

In the case of oseltamivir binding, the H274Y mutation shifted the E276 conformation closer to R292 (Fig. 3A, with representative structures in Figs. 2D–E) and the carboxyl group of the inhibitor. In contrast, the E277-R292 distance distribution remained largely unchanged (Fig. 3C). Despite the observed strengthening of electrostatic interactions between E276 and R292, an analysis of the component residue energies calculated using MM-GBSA (ΔΔ*G* ^MM-GBSA^) between the WT and H274Y trajectories showed that the mutation leads to destabilizing ΔΔ*G* contributions of +1.2 kcal/mol and +0.9 kcal/mol for E277 and E276, respectively (Table S1). While these MM-GBSA calculations were not quantitatively accurate, they were consistent with the results from the SRSM/HREX calculations and suggest that these subtle conformational changes among charged residues may disrupt oseltamivir binding.

**Figure 4:**
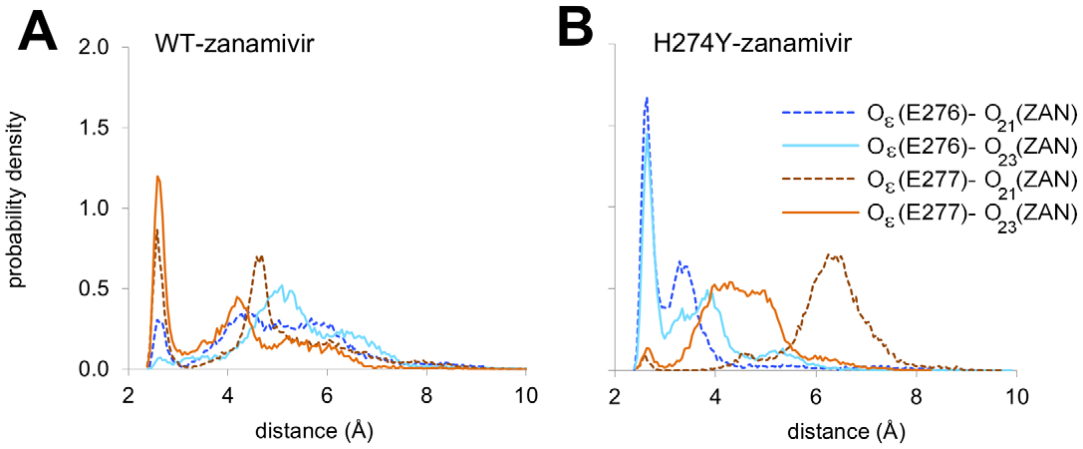
Differences in hydrogen bonding with the zanamivir tail in the H274Y mutant. Distribution of the average distance between the atoms O_ε_ of E276 and E277 with O_21_ and O_23_ in the trihydroxypropyl group of zanamivir in wild type (**A**) and in the H274Y mutant (**B**).

In the case of zanamivir binding, the H274Y mutation also led to strengthening of the E276-R292 interaction (Figs. 3B with representative structures in Fig. 2A–B). However, this change occurred concurrently with a weakening of the E277-R292 interaction (Fig. 3D) as well as changes in hydrogen bonding between the zanamivir tail and R118, E276, and E277. Specifically, the hydrogen bonds between the hydroxyl groups of the trihydroxypropyl tail of zanamivir and E277 carboxyl group in WT were replaced with hydrogen bonds with the neighboring E276 carboxyl in the mutant, as inferred from the distance distributions for the oxygen atoms from the above-mentioned groups (Fig. 4). While the ΔΔ*G* ^MM-GBSA^ of E277 was positive at +1.9 kcal/mol, the formation of inter-molecular hydrogen bonds at E276 is reflected in a ΔΔ*G* ^MM-GBSA^ of E276 that was stabilizing at −2.4 kcal/mol (Table S1).

Overall, the H274Y mutation led to subtle rearrangements of the charged residues E276, E277, and R292 within the binding pocket (see SI Section 3 in Text S1 for details). In the case of zanamivir binding, there was also a shift in hydrogen bonding between the glycerol tail and E276 and E277 that appears to, at least in part, compensate for these rearrangements. The numerous additional inter-molecular electrostatic interactions with zanamivir, such as R118-C4 carboxyl (see Figs. 2A–B and 2D–E), may also play a role in the differential resistance between oseltamivir and zanamivir observed for this mutation.

#### The N294S mutant

**Figure 5:**
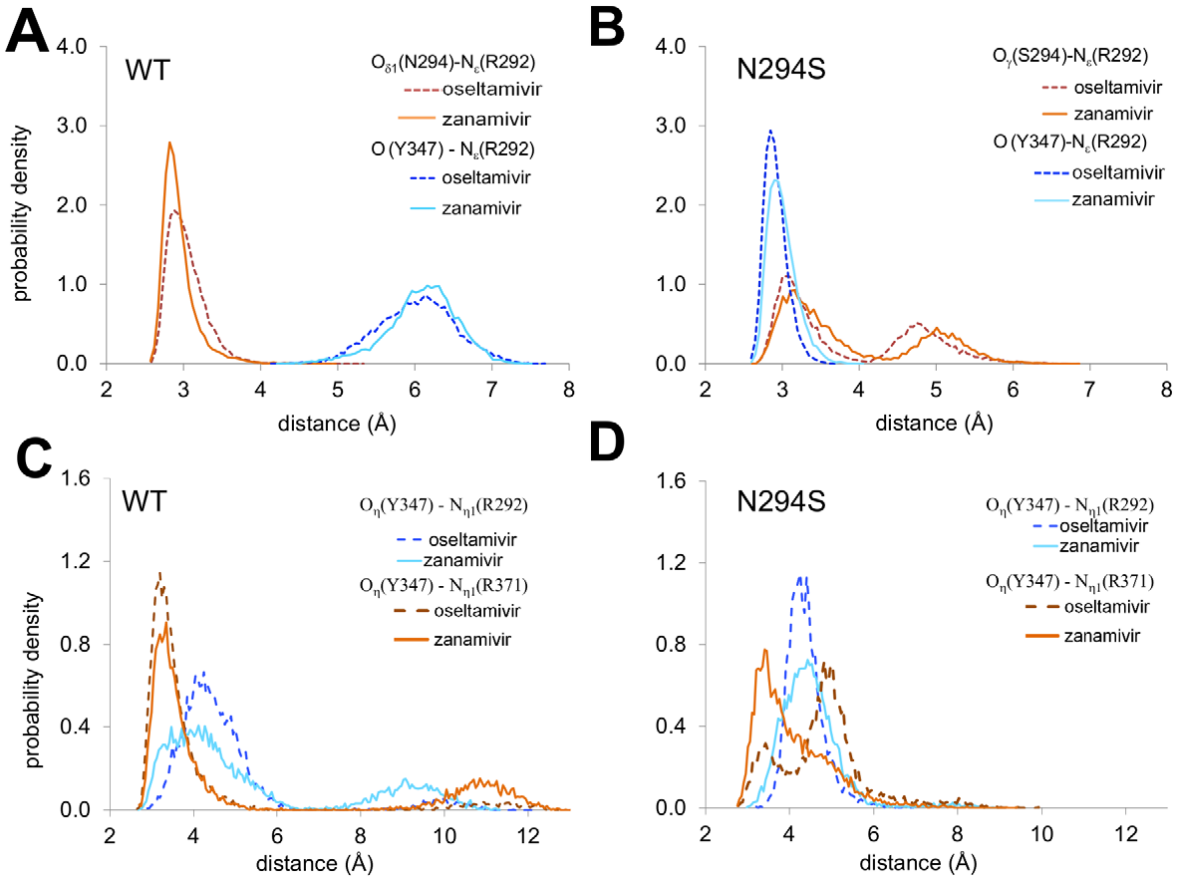
Comparison of WT and N294S mutant dynamics. (**A**) Distance distributions between the R292 N_ε_ atom and N294 O_δ1_ and Y347 carbonyl O atoms computed from SRSM/HREX simulations of the WT enzyme in complex with oseltamivir and zanamivir. (**B**) Distance distributions between the N_ε_ atom of R292 and S294 O_γ_ and Y347 carbonyl O atoms computed from simulations of the complexes of the N294S mutant with the two drugs. (**C**) Distributions of distances between the Y347 O_η_ atom and N_η1_ atoms from R292 and R371 computed from SRSM simulations of the WT enzyme in complexes with the two drugs and (**D**) for the complexes of the N294S mutant with oseltamivir and zanamivir.

SSRM/HREX simulations of the N294S mutant yielded a ΔΔ*G* of +2.2 kcal/mol and +2.3 kcal/mol for zanamivir and oseltamivir, respectively. In agreement with crystal structures of the N294S mutant, we found that the most prominent differences compared with WT was the formation of a hydrogen bond between the side chains of S294 and E276 and a flip of the main chain carbonyl of Y347. In the WT enzyme, the N294 side chain forms a hydrogen bond with the R292 side chain (Fig. 5A). In the N294S mutant, the R292 side chain predominantly interacts with the flipped Y347 carbonyl oxygen, forming a persistent hydrogen bond (Fig. 5B) that alters R292 dynamics.

In the case of oseltamivir binding, rearrangements in the N294S mutation were largely limited to the C1 carboxyl binding region between residues R292 and Y347 (representative structure shown in Fig. 2F). There were no significant rearrangements in the binding pocket beyond a slight shift in E276 to accommodate the formation of the S294-E276 hydrogen bond. MM-GBSA analysis of the binding site residues showed that R292 had a ΔΔ*G* ^MM-GBSA^ of −2.7 kcal/mol, potentially reflecting the formation of a new hydrogen bond with the Y347 carbonyl group. The lack of significant structural or energetic changes among the intermolecular interactions suggests that entropic considerations may play a major role in the disruption of oseltamivir binding observed in the N294S simulations.

MM-GBSA calculations for the zanamivir-WT complex suggested that N294 contributed, at least weakly, to inhibitor binding (Fig. S3 in Text S1), with a residue Δ*G* ^MM-GBSA^ of −1.4 kcal/mol (Table S1). The mutation of N294 to the smaller serine residue resulted in some rearrangements of the charged residues interacting with the polar tail, E276 and E277, as well as R292 and Y347, which interact with the C1 carboxyl group. Beyond the formation of the Y347-R292 hydrogen bond and the loss of the N294-R292 hydrogen bond, there were subtle shifts in the residue conformations of E276, E277, and R292. Specifically, the E276-R292 interaction was stronger, albeit mildly, and the E277-R292 interaction was weaker compared to WT (Figs. 3B and 3D, with representative structures in Figs. 2D and 2F), as was observed for the H274Y mutation. Overall, the changes involving S294 interactions were reflected in a ΔΔ*G* ^MM-GBSA^ for S294 of +1.1 kcal/mol (Table S1), suggesting that part of the weakening in zanamivir binding was directly attributable to the mutated residue itself.

#### The Y252H mutant

The Y252H mutation is classified as neutral with respect to zanamivir and confers increased sensitivity to oseltamivir. Therefore, it serves as a useful control for comparing resistant and non-resistant mutations. Our SRSM/HREX simulations yielded a ΔΔ*G* of +0.6 kcal/mol and +0.7 kcal/mol for zanamivir and oseltamivir, respectively, with no significant difference in structure between the Y252H mutant and WT. The relative positions of E276, E277, and R292 were largely unchanged (Fig. 3) and the hydrogen bonding between the glycerol tail of zanamivir and E276 and E277 was maintained. Likewise, analysis of the MM-GBSA energy revealed no significant ΔΔ*G*s for binding site residues as a result of this mutation. These results confirm that the subtle, systematic rearrangements of charged residues in the inhibitor binding site observed in the H274Y and N294S trajectories are specific to experimentally observed drug resistance mutations.

#### Testing previous hypotheses of drug resistance mechanisms

Given the thermodynamic accuracy of the SRSM/HREX simulations, we sought to test previous hypotheses of NA drug resistance through structural analysis of simulation trajectories. Prior studies have suggested that a change in the burial of the inhibitor tail as a result of a drug resistance mutation is a primary feature of NA drug resistance [10]. Specifically, the H274Y mutation is thought to confer oseltamivir resistance by decreasing the size of the binding site cavity that interacts with its hydrophobic pentoxyl tail. We calculated the change in buried surface area (BSA) corresponding to the inhibitor tail and the binding site for each of the NA mutants. Our analysis revealed no significant changes in BSA (see SI Table S2). Additionally, in the N294S mutation, the hydrogen bond formed between S294 and E276 is thought to negatively affect the hydrophobic interactions with the tail of oseltamivir [10]. While our simulations confirmed the formation of an S294-E276 hydrogen bond, the mean BSA of the oseltamivir tail (Table S2) in the mutant was largely unchanged from WT. Furthermore, no significant differences were observed in BSA in the Y252H mutation. These results suggest that systematic changes in the hydrophobic interactions with the inhibitor tail are not primarily responsible for drug resistance.

Finally, the flip of the Y347 carbonyl in the N294S mutant is believed to increase the flexibility of the Y347 side chain and weaken its interactions with the carboxyl group of oseltamivir [10]. While our simulations showed (see Fig. 5C–D) changes in the conformation of Y347 in the N294S mutant (see SI Section 4 in Text S1), there was little evidence of any interaction between Y347 and the inhibitor, suggesting that it is not directly responsible for the oseltamivir resistance observed in the simulation.

### Final remarks

We used MD simulations and statistical mechanics to quantify the effect of drug resistance mutations in NA on the ΔΔ*G* of oseltamivir and zanamivir binding. We found that implicit solvent-based methods, such as MM-GBSA, and empirical approaches, such as Rosetta, were largely unable to predict drug resistance. However, careful use of thermodynamic-integration-based approaches successfully predicted binding affinities with chemical accuracy. Ultimately, the SRSM/HREX approach yielded the most accurate and precise ΔΔ*G* values compared with those obtained experimentally. The SRSM approach minimized the degree of decoupling between the real states and the unphysical reference states, while HREX significantly enhanced conformational sampling as a result of exchanges between the TI simulation windows. Together, the SRSM/HREX approach successfully sampled a thermodynamic path between WT and mutant NA which circumvented conformational sampling barriers that significantly impeded conventional MD simulations to yield highly reliable free energy calculations. The additional computational cost associated with using HREX was practically negligible compared to SRSM because the time required for both types of runs is roughly equivalent. Finally, we must point out that the computation of ΔΔ*G*s using SRSM (or SRSM/HREX) is computationally demanding. To evaluate the six ΔΔ*G* values for NA with their corresponding standard errors, we were required to carry out a minimum of 36 runs, each 4ns-long and involving 31 replicas, for an aggregate simulation time of ∼4.5 µs. The whole analysis presented here required over 15 µs of aggregated MD simulations.

We analyzed trajectories from the SRSM/HREX simulations in order to identify the structural and energetic mechanisms underlying the computed ΔΔ*G*s. We identified a number of subtle, systematic, rearrangements in the extensive hydrogen bonding and electrostatic interactions in the inhibitor binding site in the drug resistant H274Y and N294S mutations that were largely absent in the drug-sensitive Y252H mutation. Although the exact nature of these electrostatic rearrangements varied for each drug and mutation, we hypothesize that these rearrangements in the binding pocket form the basis of drug resistance in NA. This is in contrast with the previous interpretations of the experimental structures that suggested changes in the size and hydrophobicity of the binding pocked as the primary mechanism for resistance [10].

Our study marks the most extensive use to date of molecular dynamics and thermodynamic integration on a large, pharmaceutically relevant system and demonstrates that a rigorous, computationally intensive approach can be successfully applied to studying the thermodynamic mechanisms underlying protein function that can elude traditional structure-based crystallography approaches.

## Materials and Methods

Coordinates of the protein systems were derived from the crystal structures of NA (PDB codes: 2HTY, 3CL0, 3CL2, and 3CKZ) [10]. A detailed description of the setup is provided in SI Section 1*b* in Text S1.

We used SRTI to calculate the relative free energy difference between a RS and a given end state of a system. Unless stated otherwise, all the simulation details were the same as described previously [31]. The end states in our simulations were NA variants, either free or bound to an inhibitor. To enhance sampling between the states, we employed HREX.

To run the MD simulations, we employed the GROMACS program version 4.0.5. Production runs were 4 ns long for each SRTI window and the coordinates of the system were recorded every 500 steps for subsequent analyses. SRTI simulations augmented with HREX were run using *m* = 31 windows and replica exchanges were attempted every 500 MD steps. A total of 4,000 attempted exchanges were produced, which resulted in 4 ns-long simulations per window.

### Single reference multiple mutants approach

The SRMM approach (Fig. 1) allows for simultaneous comparison of binding free energy changes between all pairs of proteins and ligands. To implement this approach, we designed a common RS for all proteins and ligands for the bound and unbound state. Portions of all three mutating residues and the ligand were decoupled in these simulations. The details are provided in SI Section 1*i* and Fig. S2A in Text S1.

### Single reference single mutant approach

The SRSM approach (Fig. 1) computed ΔΔ*G* between WT and a specific mutant for each ligand. To implement this approach we constructed specific reference states for each mutant and ligand in the bound and unbound state. Only a single amino acid was decoupled in these simulations. The details are available in SI Section 1*j* and Fig. S2B in Text S1.

### Estimation of binding affinity using the MM-PBSA/GBSA method

The MM-PBSA/GBSA method [14], as implemented in Amber10, was used to obtain additional estimates of the changes in binding free energy based on SRSM trajectories. Additional details are provided in SI Section 1*k* in Text S1.

### Estimation of the binding affinity using Rosetta

RosettaInterface [32] uses computational mutagenesis to predict the change in binding free energy of a protein-protein interaction associated with point mutations. Details on the implementation of RosettaInterface for protein-ligand interactions are provided in SI Section 1*l* in Text S1.

## Supporting Information

Table S1 Energy decomposition analysis of WT, and H274Y, N294S and Y252H mutants.
(PDF)

Table S2 Average buried surface area of the pentoxyl substituent of oseltamivir.
(PDF)

Text S1 Supplemental information file contains an extended Materials and Methods section with a detailed description of the techniques and protocols used in the research. Additional discussions of the key NA-inhibitor interactions, the H274Y and N294S mutants are provided. The file contains supplemental figures S1 to S5 with their respective legends, and five appendices.
(PDF)
