# Supplementary material for: Quantitative Predictions of Binding Free Energy Changes in Drug-Resistant Influenza Neuraminidase
Source: PLoS Comput Biol. 2012 Aug 30;8(8):e1002665. doi: 10.1371/journal.pcbi.1002665 (PMC3431292; doi:10.1371/journal.pcbi.1002665)
Supplement: Table S1 — Energy decomposition analysis of WT, and H274Y, N294S and Y252H mutants. (PDF) [file pcbi.1002665.s001.pdf]

Table S1: Energy decomposition analysis. Residue contributions for complexes of wild type (WT) and N294S mutant with oseltamivir and zanamivir

Residue	N294S $\Delta G^{MM\text{-}GBSA}$ (kcal/mol)	WT $\Delta G^{MM\text{-}GBSA}$ (kcal/mol)	$\Delta\Delta G^{MM\text{-}GBSA}$ (kcal/mol)
ARG-118	0.1 (1.1)	-0.1(1.0)	0.2(1.5)
GLU-119	-5.1 (1.3)	-5.6(0.9)	0.5(1.6)
LEU-134	0.0 (0.1)	-0.1(0.0)	0.0(0.1)
ASP-151	-3.0 (1.0)	-2.9(0.8)	-0.1(1.3)
ARG-152	-1.1 (0.9)	-0.7(0.5)	-0.4(1.1)
ARG-156	1.8 (0.8)	2.0(0.5)	-0.3(1.0)
TRP -178	-0.6 (0.3)	-0.8(0.3)	0.2(0.4)
SER-179	-0.6 (0.2)	-0.6(0.1)	0.0(0.2)
ILE-222	-1.0 (0.2)	-1.0(0.1)	0.0(0.3)
ARG-224	-0.4 (0.4)	-0.2(0.2)	-0.2(0.4)
GLU-227	-0.8 (0.9)	-1.4(0.7)	0.6(1.2)
SER-246	-0.1 (0.2)	-0.2(0.1)	0.1(0.2)
HIS-274	0.0 (0.1)	0.1(0.0)	-0.1(0.1)
GLU-276	-0.5 (0.3)	-0.8(0.2)	0.3(0.4)
GLU-277	-0.8 (0.8)	-1.2(0.5)	0.4(1.0)
ARG-292	-7.2 (1.3)	-4.5(1.1)	-2.7(1.7)
ASN-294*	-0.2 (0.2)	-0.3(0.1)	0.1(0.2)
TYR-347	-0.0 (0.3)	0.1(0.2)	-0.1(0.4)
GLY-348	-0.1 (0.1)	-0.0(0.0)	-0.1(0.1)
VAL-349	-0.5 (0.1)	-0.4(0.1)	-0.1(0.2)
LYS-350	0.1 (0.1)	0.1(0.1)	-0.0(0.1)
ARG-371	-7.8 (1.1)	-6.8(0.9)	-1.0(1.5)
TYR-406	-1.6 (0.4)	-1.9(0.3)	0.3(0.5)
oseltamivir	-28.9 (2.7)	-26.4(1.9)	-2.5(3.3)

Residue	N294S $\Delta G^{MM\text{-}GBSA}$ (kcal/mol)	WT $\Delta G^{MM\text{-}GBSA}$ (kcal/mol)	$\Delta\Delta G^{MM\text{-}GBSA}$ (kcal/mol)
ARG-118	-4.2 (2.1)	-5.0(1.0)	0.8(2.3)
GLU-119	-1.8 (1.3)	-2.2(0.7)	0.4(1.5)
LEU-134	-0.3 (0.1)	-0.3(0.1)	0.0(0.1)
ASP-151	0.0 (0.6)	-0.1(0.3)	0.1(0.7)
ARG-152	-2.5 (1.1)	-2.4(0.6)	-0.1(1.2)
ARG-156	1.2 (0.8)	1.5(0.4)	-0.3(0.9)
TRP-178	-1.8 (0.8)	-2.1(0.5)	0.4(0.9)
SER-179	-0.6 (0.4)	-0.6(0.2)	-0.0(0.5)
ILE-222	-0.8 (0.2)	-0.8(0.1)	-0.0(0.3)
ARG-224	-1.4 (0.8)	-1.4(0.4)	0.0(0.9)
GLU-227	-4.4 (1.8)	-4.5(0.8)	0.0(2.0)
SER-246	-0.4 (0.4)	-0.3(0.2)	-0.0(0.4)
HIS-274	-0.2 (0.2)	-0.1(0.1)	-0.1(0.2)
GLU-276	-0.2 (1.1)	-0.8(0.7)	0.6(1.3)
GLU-277	-2.7 (1.8)	-3.4(1.0)	0.7(2.0)
ARG-292	-5.9 (2.1)	-5.0(0.9)	-0.9(2.3)
ASN-294*	-0.3 (0.4)	-1.4(0.3)	1.1(0.5)
TYR-347	0.0 (0.3)	-0.0(0.1)	-0.0(0.3)
GLY-348	-0.2 (0.1)	-0.2(0.0)	0.0(0.1)
VAL-349	-0.5 (0.2)	-0.5(0.1)	0.0(0.2)
LYS-350	0.2 (0.3)	0.1(0.1)	0.1(0.3)
ARG-371	-6.8 (1.3)	-6.7(0.7)	-0.1(1.5)
TYR-406	-1.6 (0.6)	-1.7(0.3)	0.1(0.7)
zanamivir	-20.9 (3.6)	-24.3(2.1)	3.4(4.1)

Table S1 *(cont.)*: Energy decomposition analysis. Residue contributions for complexes of WT and H274Y mutant with oseltamivir and zanamivir

Residue	H274Y $\Delta G^{MM\text{-}GBSA}$ (kcal/mol)	WT $\Delta G^{MM\text{-}GBSA}$ (kcal/mol)	$\Delta\Delta G^{MM\text{-}GBSA}$ (kcal/mol)
ARG-118	-0.3 (1.8)	-0.1(1.0)	-0.2(2.0)
GLU-119	-4.8 (1.6)	-5.6(0.9)	0.8(1.8)
LEU-134	-0.1 (0.1)	-0.1(0.0)	0.0(0.1)
ASP-151	-2.2 (1.1)	-2.9(0.8)	0.7(1.4)
ARG-152	-1.7 (0.9)	-0.7(0.5)	-1.0(1.1)
ARG-156	1.5 (0.7)	2.0(0.5)	-0.5(0.9)
TRP-178	-0.7 (0.3)	-0.8(0.3)	0.1(0.4)
SER-179	-0.6 (0.2)	-0.6(0.1)	0.0(0.2)
ILE-222	-1.1 (0.2)	-1.0(0.1)	-0.0(0.2)
ARG-224	-0.8 (0.4)	-0.2(0.2)	-0.6(0.4)
GLU-227	-1.5 (1.3)	-1.4(0.7)	-0.1(1.5)
SER-246	0.0 (0.2)	-0.2(0.1)	0.2(0.2)
HIS-274*	-0.2 (0.1)	0.1(0.0)	-0.3(0.1)
GLU-276	0.4 (0.5)	-0.8(0.2)	1.2(0.6)
GLU-277	-0.3 (0.9)	-1.2(0.5)	0.9(1.0)
ARG-292	-5.0 (2.0)	-4.5(1.1)	-0.5(2.3)
ASN-294	-0.3 (0.2)	-0.3(0.1)	0.0(0.2)
TYR-347	0.1 (0.3)	0.1(0.2)	-0.0(0.3)
GLY-348	0.0 (0.1)	-0.0(0.0)	-0.0(0.1)
VAL-349	-0.4 (0.1)	-0.4(0.1)	0.0(0.2)
LYS-350	0.1 (0.1)	0.1(0.1)	0.0(0.1)
ARG-371	-7.1 (1.0)	-6.8(0.9)	-0.3(1.4)
TYR-406	-1.8 (0.5)	-1.9(0.3)	0.1(0.6)
oseltamivir	-25.1 (3.0)	-26.4(1.9)	1.3(3.5)

Residue	H274Y $\Delta G^{MM\text{-}GBSA}$ (kcal/mol)	WT $\Delta G^{MM\text{-}GBSA}$ (kcal/mol)	$\Delta\Delta G^{MM\text{-}GBSA}$ (kcal/mol)
ARG-118	-2.2 (1.9)	-5.0(1.0)	2.8(2.2)
GLU-119	-2.2 (1.1)	-2.2(0.7)	-0.0(1.3)
LEU-134	-0.3 (0.1)	-0.3(0.1)	0.0(0.1)
ASP-151	-0.1 (0.6)	-0.1(0.3)	-0.0(0.7)
ARG-152	-3.1 (0.8)	-2.4(0.6)	-0.7(1.0)
ARG-156	1.8 (0.7)	1.5(0.4)	0.3(0.8)
TRP-178	-1.9 (0.7)	-2.1(0.5)	0.2(0.8)
SER-179	-0.6 (0.3)	-0.6(0.2)	-0.0(0.3)
ILE-222	-0.7 (0.2)	-0.8(0.1)	0.1(0.2)
ARG-224	-1.3 (0.5)	-1.4(0.4)	0.1(0.6)
GLU-227	-3.7 (1.2)	-4.5(0.8)	0.8(1.4)
SER-246	-0.2 (0.3)	-0.3(0.2)	0.1(0.4)
HIS-274*	-0.2 (0.1)	-0.1(0.1)	-0.1(0.1)
GLU-276	-3.3 (1.3)	-0.8(0.7)	-2.4(1.5)
GLU-277	-1. 5 (1.6)	-3.4(1.0)	1.9(1.9)
ARG-292	-6.5 (1.5)	-5.0(0.9)	-1.5(1.8)
ASN-294	-0.9 (0.5)	-1.4(0.3)	0.5(0.6)
TYR-347	0.0 (0.3)	-0.0(0.1)	0.0(0.3)
GLY-348	-0.2 (0.1)	-0.2(0.0)	0.0(0.1)
VAL-349	-0.5 (0.1)	-0.5(0.1)	0.0(0.2)
LYS-350	0.2 (0.1)	0.1(0.1)	0.1(0.2)
ARG-371	-7.1 (0.8)	-6.7(0.7)	-0.4(1.1)
TYR-406	-1.4 (0.5)	-1.7(0.3)	0.3(0.6)
zanamivir	-21.7 (3.7)	-24.3(2.1)	2.7(4.3)

Table S1 *(cont.)*: Energy decomposition analysis. Residue contributions for complexes of WT and Y252H mutant with oseltamivir and zanamivir

Residue	Y252H $\Delta G^{MM\text{-}GBSA}$ (kcal/mol)	WT $\Delta G^{MM\text{-}GBSA}$ (kcal/mol)	$\Delta\Delta G^{MM\text{-}GBSA}$ (kcal/mol)
ARG-118	0.1 (1.2)	-0.1(1.0)	0.2(1.5)
GLU-119	-5.0 (1.2)	-5.6(0.9)	0.6(1.5)
LEU-134	-0.1 (0.1)	-0.1(0.0)	0.0(0.1)
ASP-151	-3.2 (1.0)	-2.9(0.8)	-0.3(1.2)
ARG-152	-0.7 (0.7)	-0.7(0.5)	0.0(0.9)
ARG-156	1.8 (1.0)	2.0(0.5)	-0.2(1.1)
TRP-178	-0.6 (0.2)	-0.8(0.3)	0.2(0.4)
SER-179	-0.6 (0.2)	-0.6(0.1)	0.0(0.2)
ILE-222	-0.9 (0.2)	-1.0(0.1)	0.1(0.2)
ARG-224	-0.3 (0.4)	-0.2(0.2)	-0.1(0.4)
GLU-227	-0.7 (0.8)	-1.4(0.7)	0.7(1.0)
SER-246	-0.3 (0.2)	-0.2(0.1)	-0.0(0.2)
HIS-274	0.2 (0.1)	0.1(0.0)	0.1(0.1)
GLU-276	-0.8 (0.4)	-0.8(0.2)	-0.0(0.4)
GLU-277	-1.1 (0.9)	-1.2(0.5)	0.2(1.0)
ARG-292	-5.5 (1.5)	-4.5(1.1)	-1.0(1.9)
ASN-294	-0.3 (0.2)	-0.3(0.1)	0.0(0.2)
TYR-347	0.2 (0.3)	0.1(0.2)	0.1(0.4)
GLY-348	0.0 (0.1)	-0.0(0.0)	-0.0(0.1)
VAL-349	-0.4 (0.1)	-0.4(0.1)	0.0(0.2)
LYS-350	0.1 (0.1)	0.1(0.1)	-0.0(0.2)
ARG-371	-6.9 (1.7)	-6.8(0.9)	-0.1(1.9)
TYR-406	-1.8 (0.5)	-1.9(0.3)	0.1(0.6)
oseltamivir	-27.5 (2.5)	-26.4(1.9)	-1.1(3.2)

Residue	Y252H $\Delta G^{MM\text{-}GBSA}$ (kcal/mol)	WT $\Delta G^{MM\text{-}GBSA}$ (kcal/mol)	$\Delta\Delta G^{MM\text{-}GBSA}$ (kcal/mol)
ARG-118	-4.3 (2.0)	-5.0(1.0)	0.7(2.3)
GLU-119	-1.8 (1.2)	-2.2(0.7)	0.4(1.4)
LEU-134	-0.4 (0.1)	-0.3(0.1)	-0.0(0.2)
ASP-151	-0.2 (0.8)	-0.1(0.3)	-0.1(0.8)
ARG-152	-2.1 (1.1)	-2.4(0.6)	0.3(1.3)
ARG-156	1.8 (0.7)	1.5(0.4)	0.3(0.8)
TRP-178	-2.7 (0.7)	-2.1(0.5)	-0.6(0.9)
SER-179	-0.8 (0.3)	-0.6(0.2)	-0.2(0.4)
ILE-222	-0.8 (0.2)	-0.8(0.1)	0.0(0.2)
ARG-224	-1.8 (0.7)	-1.4(0.4)	-0.3(0.8)
GLU-227	-4.0 (1.2)	-4.5(0.8)	0.5(1.5)
SER-246	-0.5 (0.4)	-0.3(0.2)	-0.2(0.5)
HIS-274	-0.1 (0.1)	-0.1(0.1)	-0.0(0.2)
GLU-276	-0.1 (1.1)	-0.8(0.7)	0.7(1.3)
GLU-277	-2.0 (1.6)	-3.4(1.0)	1.4(1.9)
ARG-292	-5.6 (1.8)	-5.0(0.9)	-0.6(2.0)
ASN-294	-1.3 (0.6)	-1.4(0.3)	0.1(0.7)
TYR-347	-0.1 (0.2)	-0.0(0.1)	-0.1(0.2)
GLY-348	-0.2 (0.1)	-0.2(0.0)	0.0(0.1)
VAL-349	-0.5 (0.2)	-0.5(0.1)	0.0(0.2)
LYS-350	0.0 (0.2)	0.1(0.1)	-0.1(0.2)
ARG-371	-6.3 (1.8)	-6.7(0.7)	0.4(2.0)
TYR-406	-1.5 (0.5)	-1.7(0.3)	0.2(0.6)
zanamivir	-24.6 (4.1)	-24.3(2.1)	-0.3(4.6)

$\Delta G^{MM\text{-}GBSA}$ values are reported by the Molecular Mechanics - Generalized Born Surface Area (MM-GBSA) program as TGBTOT. $\Delta G^{MM\text{-}GBSA}$ values included those contributions described in equation (7) (see SI Section "*Estimation of the binding affinity using MM-PBSA/GBSA methods*") with the exception of the entropic terms (1). Residues for which the absolute value of $\Delta G^{\#}$ exceeded 0.2 kcal/mol in at least one calculation are included. Standard deviations are shown in parentheses. The symbol '*' is used to mark residues that are mutated.
