# Supplementary material for: Quantitative Predictions of Binding Free Energy Changes in Drug-Resistant Influenza Neuraminidase
Source: PLoS Comput Biol. 2012 Aug 30;8(8):e1002665. doi: 10.1371/journal.pcbi.1002665 (PMC3431292; doi:10.1371/journal.pcbi.1002665)
Supplement: Table S2 — Average buried surface area of the pentoxyl substituent of oseltamivir. (PDF) [file pcbi.1002665.s002.pdf]

Table S2: Average buried surface area of (*a*) the pentoxyl substituent of oseltamivir (column 2) and (*b*) the group of atoms involving the pentoxyl substituent of oseltamivir and hydrophobic carbons from the side chains in the active site that interact with that substituent (column 3). Values were computed with the program *g_sas* (2) provided with GROMACS using the trajectories for the SRSM-HREX approach. Standard deviations are shown in parentheses.

Molecule	Buried Surface Area Pentoxyl group (nm^2)	Buried Surface Area Site + Pentoxyl group (nm^2)
WT	0.2(0.2)	0.5(0.2)
H274Y	0.3(0.2)	0.5(0.2)
N294S	0.4(0.2)	0.7(0.2)
Y252H	0.3(0.2)	0.7(0.2)
