# Supplementary material for: Quantitative Predictions of Binding Free Energy Changes in Drug-Resistant Influenza Neuraminidase
Source: PLoS Comput Biol. 2012 Aug 30;8(8):e1002665. doi: 10.1371/journal.pcbi.1002665 (PMC3431292; doi:10.1371/journal.pcbi.1002665)
Supplement: Text S1 — Supplemental information file contains an extended Materials and Methods section with a detailed description of the techniques and protocols used in the research. Additional discussions of the key NA-inhibitor interactions, the H274Y and N294S mutants are provided. The file contains supplemental figures S1 to S5 with their respective legends, and five appendices. (PDF) [file pcbi.1002665.s003.pdf]

Text S1

Supporting Information for

Quantitative predictions of binding free energy changes in drug-resistant influenza neuraminidase

Daniel R. Ripoll[†], Ilja V. Khavrutskii[†], Sidhartha Chaudhury[†], Jin Liu[†], Robert A. Kuschner[‡], Anders Wallqvist[†], Jaques Reifman[†*]

[†]DoD Biotechnology High Performance Computing Software Applications Institute, Telemedicine and Advanced Technology Research Center, US Army Medical Research and Materiel Command, Fort Detrick, MD 21702

[‡]Walter Reed Army Institute of Research, Emerging Infectious Diseases Research Unit, 503 Robert Grant Avenue, Silver Spring, MD 20910, USA

[*]To whom correspondence should be addressed: Jaques Reifman, DoD Biotechnology High Performance Computing Software Applications Institute, Telemedicine and Advanced Technology Research Center, US Army Medical Research and Materiel Command, Fort Detrick, MD 21702, Phone: (301) 619-8130, e-mail: jaques.reifman@us.army.mil

Contents

1. *Materials and Methods* S3
    - a. *Ligand parameters* S3
    - b. *System setup* S6
    - c. *Molecular dynamics simulations* S6
    - d. *Thermodynamic integration setup* S7
    - e. *Reference states for SRTI simulations* S7
    - f. *Regular SRTI simulations* S10
    - g. *HREX-SRTI simulations* S10
    - h. *Estimation of free energy changes from experimental measurements* S10
    - i. *Single reference multiple mutants (SRMM) approach* S11
    - j. *Single reference single mutant (SRSM) approach* S12
    - k. *Estimation of the binding affinity using the MM/PBSA method* S12
    - l. *Estimation of the binding affinity using RosettaInterface* S14
2. *Key NA-inhibitor interactions* S15
3. *The H274Y mutant: Constraints on the χ_1 dihedral angle of E276* S17
4. *The N294S mutant: The Y347 side chain* S21
5. *References* S23
6. *Appendix 1: mol2 file for oseltamivir* S26
7. *Appendix 2: mol2 file for zanamivir* S28
8. *Appendix 3: mol2 file for core-ligand* S30
9. *Appendix 4: mapping files for SRMM calculations* S31
10. *Appendix 5: mapping files for SRSM calculations* S35

1. Materials and Methods

a. Ligand parameters

The General AMBER Force Field (GAFF)[1] parameters for oseltamivir, zanamivir, and their common reference (Fig. S1A-B) were derived as described in previous publications [2,3] and outlined below. Starting from Simplified Molecular Input Line Entry System (SMILES) strings with proper stereochemical and protonation (zwitterionic) state information [4,5], we employed the OMEGA2 program [6,7] to generate the initial coordinates of inhibitors. The same program was used to sample the configurational space of each molecule by generating a maximum of 99 conformations within 20 kcal/mol of each other. Each conformation from the initial ensemble was then geometrically optimized using the Austin Model 1 (AM1) semi-empirical quantum mechanical potential [8] as implemented in the public domain MOPAC7, version 1.11 [9].

The molecules considered here are flexible and must be described by a set of unique conformations to derive conformation-independent partial charges. Optimization of zwitterionic molecules in gas phase often presents a problem due to spurious proton transfers that change the connectivity of atoms within the molecule and quench the charge separation. While zanamivir behaved well during optimization, likely due to the larger delocalization of the positive charge on its guanidinium group, certain conformations of oseltamivir experienced proton transfer from ammonium to the carboxyl group. Conformations resulting from undesirable proton transfers were identified and removed based on energy criteria and visual inspection. Unique conformations were then identified using energy and mass weighted best-fit root mean squared deviations (RMSD) criteria excluding symmetric atoms. Specifically, energies from MOPAC calculations were first converted into Boltzmann weights at 300K. All the conformations were grouped by their weights using a threshold of 10^{-6}, and groups were sorted by weights.

Furthermore, RMSDs were scaled by the inverse of the square root of the number of atoms involved. For example, in oseltamivir the twin atoms of the hydrophobic tail, the carboxyl oxygen atoms, the hydrogen atoms of the ammonia and the methyl groups, and the two methylene hydrogens of the six-membered ring were excluded from the RMSD calculation, leaving 21 atoms out of a total of 44. Within the same weight group, two conformations were considered different if their scaled RMSD exceeded 0.001 nm. Groups with different weights were considered distinct if the lowest scaled RMSD between representative conformations exceeded 0.005 nm. In cases where two groups were deemed identical, the group with the largest Boltzmann weight was retained. Degeneracy of the remaining groups was determined by the number of unique conformers within the group.

The AM1 partial charges from each unique conformation were accumulated into a conformation-independent set of charges using Boltzmann weighting by their AM1 energies at the target temperature of 300 K with appropriate degeneracies. The resulting AM1 charges were symmetrized where applicable and then augmented through the Bond Charge Correction (BCC) procedure [8,10] implemented within the ANTECHAMBER program [11,12] distributed with AMBER TOOLS, version 1.2. The resulting set of AM1BCC charges is expected to reproduce HF/6-31G* RESP charges with good approximation [13,14,15,16].

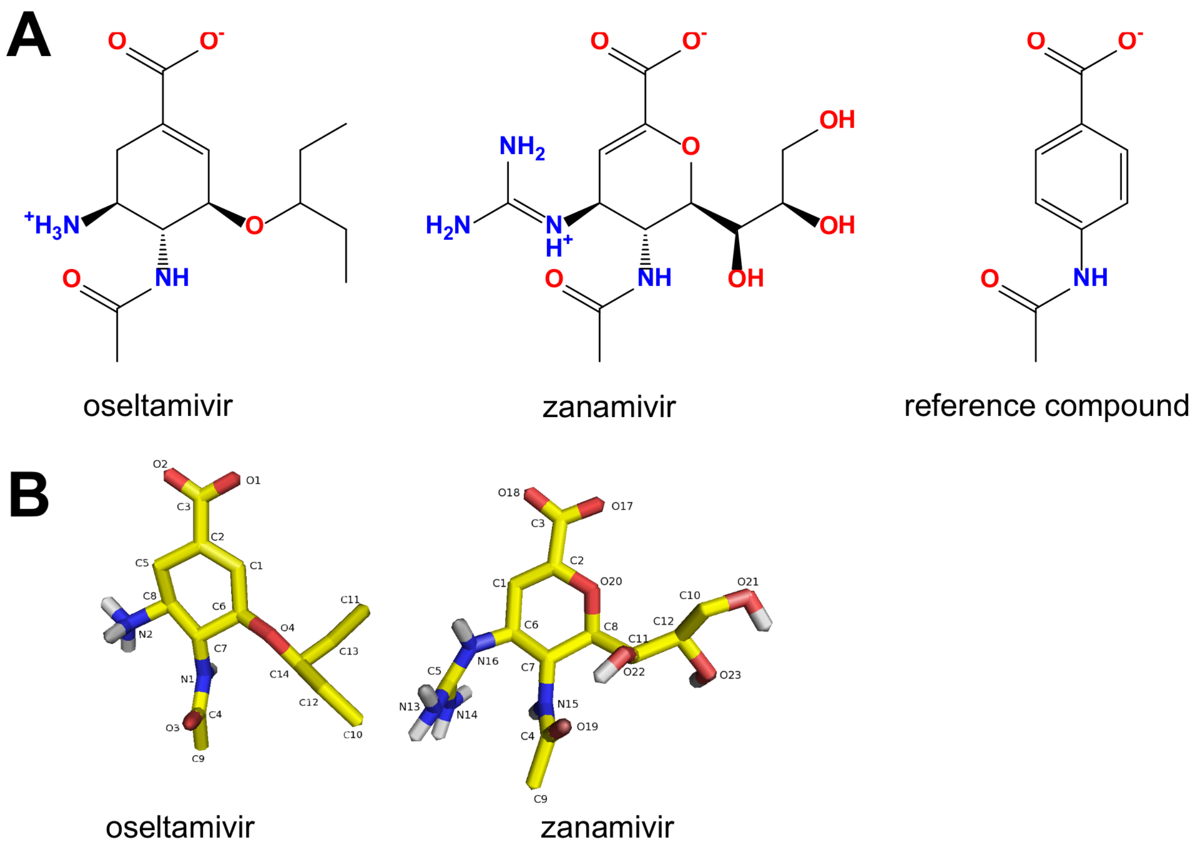

Figure S1: (**A**) Schematic representation of oseltamivir, zanamivir, and the common reference compound. (**B**) Atom-naming and numbering convention for oseltamivir and zanamivir. Heavy and polar hydrogen atoms are shown using a "stick" model. Carbon, nitrogen, oxygen, and hydrogen atoms are colored yellow, blue, red, and white, respectively.

b. System setup

The protein systems used in the simulations were derived from the crystal structures of N1 neuraminidase (NA) and its mutants (PDB codes: 2HTY, 3CL0, 3CL2, and 3CKZ) [17]. The program REDUCE [18] was used to add hydrogen atoms and to determine the protonation states of the histidine residues. To optimize hydrogen-bonded networks, 180 degree rotations of the Asn, Gln, and His residues were allowed. All the His residues were then visually inspected along with residues within a 5 Å radius to confirm their protonation states. Finally, disulfide bonds were specified to complete the protein system setup. Solvation effects were modeled using a periodic cubic box of explicit three-point transferable inter-molecular potential (TIP3P) water molecules that provided at least 10 Å-thick padding of the solute. The systems were solvated using the LEAP program from the AMBER TOOLS [19].

c. Molecular dynamics simulations

Production runs were performed in the NPT ensemble at temperature T = 300 K and pressure P = 1 atm, following an equilibration protocol described in our previous work [2]. The program GROMACS version 4.0.5 (single precision) was used to run all the simulations. For temperature and pressure control, we employed Langevin thermostat and Berendsen barostat [20,21,22,23] with identical collision frequencies of 2 ps^{-1}. All bonds involving hydrogen atoms were constrained throughout the simulations using LINCS [24] and the integration time step was set to 2 fs. The Particle Mesh Ewald (PME) approach was used to compute the electrostatics [20,21,22,23] with a 1 nm real space cutoff. We used the same cutoff for the van der Waals interactions that were switched off for the range between 0.8 and 0.9 nm.

d. Thermodynamic Integration setup

We employed a recently developed variant of Thermodynamic Integration (TI), namely the Single Reference TI (SRTI), optionally augmented with Hamiltonian Replica Exchange (HREX) [2,3]. For all the SRTI simulations reported in this work, we used soft-core potentials with p = 1 (refer to GROMACS manual for notation used in soft-core potentials) and previously optimized values [3,25] of $\alpha = 0.4$ and $\sigma = 0.25$.

Each SRTI simulation employed identical starting configurations across all windows. For each window, values of $\frac{\partial V}{\partial \lambda}$ were recorded at every time step. The mean values $\left\langle \frac{\partial V}{\partial \lambda} \right\rangle$ for all the windows were assembled into the final work using the Fourier Beads integration procedure described earlier [2]. The final relative binding free energies were reported with respect to the wild type (WT) enzyme. The standard deviations were derived using at least two independent simulations in each leg of the thermodynamic cycle.

e. Reference states for SRTI simulations

The real and reference states of each system correspond to values 0 and 1 of the Hamiltonian coupling parameter λ, respectively. Based on the choice of the reference state, our calculations were split into two approaches, namely SRMM and SRSM.

In the SRMM approach, we used an artificial reference protein by itself or in complex with an artificial reference ligand. In this case, the complexes of all the mutants with all the ligands studied or the mutants by themselves can be mapped to the SRMM reference state. To achieve that, we first derived parameters for a simpler (stripped down) molecule (Fig. S1A). Unlike the inhibitors, the reference molecule was neither chiral nor zwitterionic. Furthermore, it lacked the tail and its six-membered ring did not have any oxygen atoms and was not saturated. The only

functional groups preserved from the inhibitors were the carboxyl and the amide. This special molecule provided the foundation for our unphysical reference ligand. However, many other molecules could have been used here as well. The same atoms of the reference ligand must be mapped to both inhibitors for SRTI to work. All the remaining unmapped atoms of the reference ligand would "collapse" onto the nearest mapped atoms. If all atoms of the reference molecule were mapped, then the reference state would correspond to a real state.

In devising the SRMM protein reference state suitable for mapping all of the mutants of interest in addition to WT, we looked for standard residues that would allow us to map all the heavy atoms to the residues involved in the mutations. Thus, we chose an Asn residue to map mutations involving changes of Y to H and H to Y, and a Ser residue for changing N to S. Just like in the case with the reference ligand, any unmapped atom of the reference residues was collapsed onto the nearest mapped heavy atom, producing a 'pseudo' residue. Thus, for the SRMM approach, where all three mutations needed to be mapped to the same reference protein, we used the unphysical triple mutant Y252N^p:H274N^p:N294S^p as the reference protein.

In devising the SRSM protein state, each mutation was considered separately. Therefore, the SRSM approach employed a different reference state for each mutant. The SRSM reference states corresponded to the three single mutants Y252N^p, H274N^p, and N294S^p. Note that the ligands were not mapped to any reference ligand in SRSM.

Mol2 files are provided in appendices 1-3 for the oseltamivir, zanamivir and reference core ligands with the conformation independent AM1BCC charges. Files containing the mappings of the protein residues and those of the ligands to the reference states used in alchemical transformations are also provided in appendices 4 and 5.

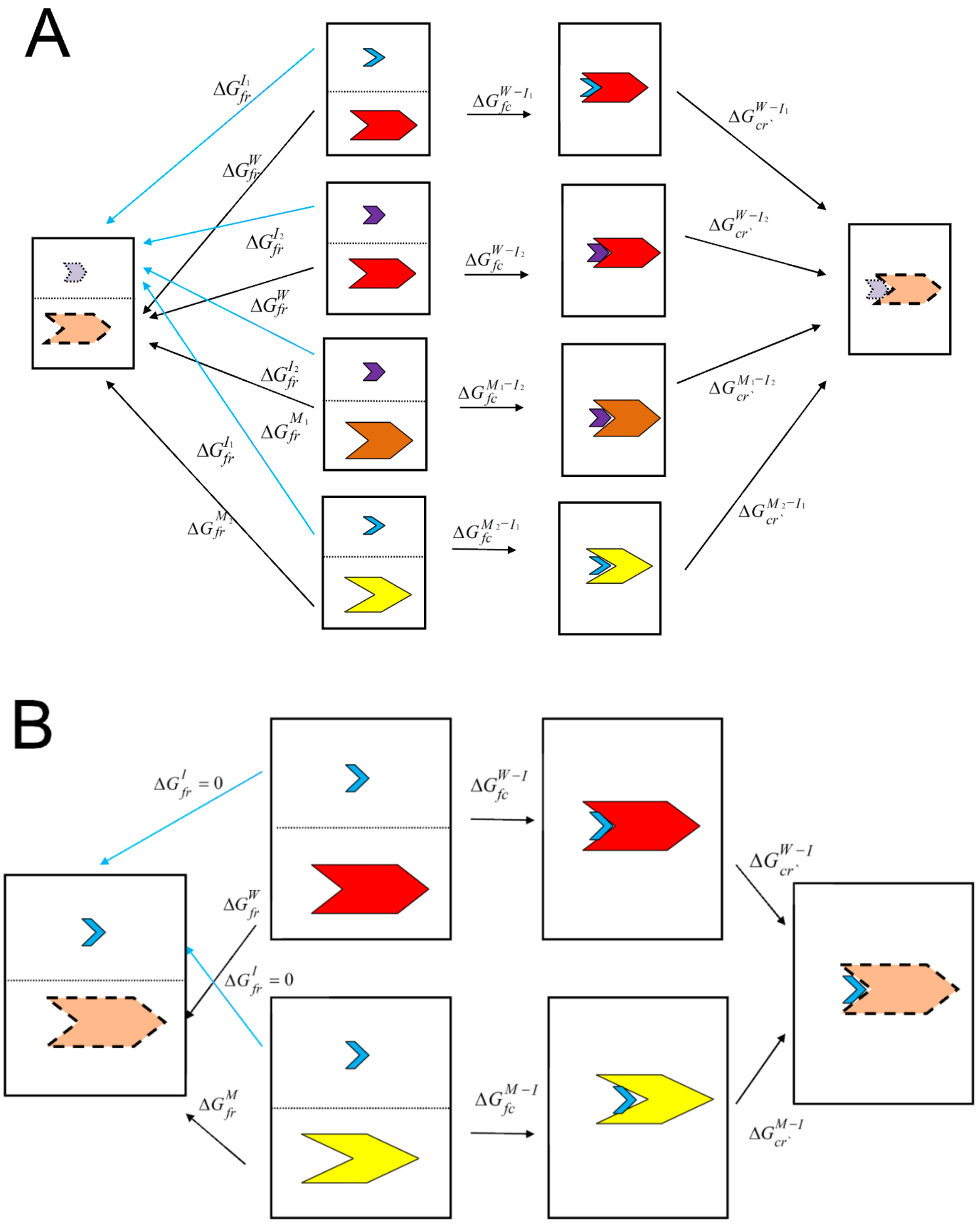

$$-\Delta G^{I}_{fr} - \Delta G^{M}_{fr} + \Delta G^{M-I}_{fc} + \Delta G^{M-I}_{cr`} = -\Delta G^{I}_{fr} - \Delta G^{W}_{fr} + \Delta G^{W-I}_{fc} + \Delta G^{W-I}_{cr`}$$

$$\Delta G^{M-I}_{fc} - \Delta G^{W-I}_{fc} = \Delta G^{M}_{fr} - \Delta G^{W}_{fr} - \Delta G^{M-I}_{cr`} + \Delta G^{W-I}_{cr`}$$

Figure S2: (**A**) Thermodynamic schema used in the SRMM approach. (**B**) Thermodynamic schema used in the SRSM approach.

f. Regular SRTI simulations

To obtain the alchemical free energies or reversible works, the corresponding SRTI transformations employed m = 16 equally separated λ-windows. Coordinates of the system were saved every 1,000 steps (2 ps).

g. HREX-SRTI simulations

To run HREX-SRTI simulations, we employed an in-house PERL script interfaced with GROMACS. Replica exchanges were attempted every 500 steps (1 ps). For these simulations, exchanges were attempted a total of 4,000 times, resulting in 4 ns-long simulations of each window. Each HREX run was carried out using m = 31 windows. Coordinates of the system for subsequent analyses were recorded every 1 ps.

h. Estimation of free energy changes from the experimental measurements

Collins and coworkers [17] used a simple model for competitive inhibition of an enzyme (see supplemental Fig. S1 of the above-mentioned reference) to interpret the NA enzymatic activity data obtained from fluorescent measurements. Based on such a model, they derived a series of activity, binding, and kinetic parameters for N1 NA WT and mutants (listed in Table 1 of Collins et al. [17]). Among the magnitudes of interest, relative K_I values (K_I^{rel}) for three different mutants (M) with respect to wild type (W) were provided for oseltamivir and zanamivir, with

$$K_I^{rel} = \frac{K_I^M}{K_I^W} \tag{1}$$

where K_I^W values are 0.32 nM for oseltamivir and 0.1 nM for zanamivir [17]. The inhibitory constant, K_I, for a particular inhibitor is related to binding free energy, ΔG, by:

$$K_b = 1/K_I = e^{-\Delta G/RT} \quad (2)$$

where K_b is the association constant, R is the gas constant, and T the temperature in Kelvin. By combining equations (1) and (2) and performing few mathematical operations, we can obtain an experimental estimate of the change in binding affinity, $\Delta\Delta G^{W\rightarrow M}$ as:

$$\Delta\Delta G^{W\rightarrow M} = \Delta G^{M} - \Delta G^{W} = RT\ln K_I^M - RT\ln K_I^W = RT\ln K_I^{rel} \quad (3)$$

i. Single reference multiple mutants (SRMM) approach

The computation of $\Delta\Delta G$ values can be derived by using the thermodynamic cycle shown in Fig. S2A. Such an approach allows for comparison of changes in free energies between pairs of proteins (M_i, M_j, e.g., mutant and/or WT enzymes) bound to the same or different ligands (I_k, I_l), and the $\Delta\Delta G^{M_i\rightarrow M_j}(I_k, I_l)$ values can be computed from the relation:

$$\begin{aligned}\Delta\Delta G^{M_i\rightarrow M_j}(I_k, I_l) &= \Delta G_{fc}^{M_j-I_l} - \Delta G_{fc}^{M_i-I_k} \\ &= \Delta G_{fr}^{I_l} - \Delta G_{fr}^{I_k} + \Delta G_{fr}^{M_j} - \Delta G_{fr}^{M_i} + \Delta G_{cr`}^{M_i-I_k} - \Delta G_{cr`}^{M_j-I_l}\end{aligned} \quad (4)$$

where $\Delta G_{fc}^{M_j-I_l}$ is the free energy change due to the formation of the complex of the protein M_j with the ligand/inhibitor I_l, $\Delta G_{fr}^{M_j}$ is the change in free energy for the transformation of protein M_j from its *free* (*f*) state in solution to the *reference* state r, $\Delta G_{fr}^{I_l}$ is the change in free energy for the transformation of the free ligand/inhibitor I_l from its *free* state in solution to the *reference* state *r*, and $\Delta G_{cr`}^{M_j-I_l}$ is the change in free energy for the transformation of the protein $M_j$ and ligand $I_l$ in *complex* from its state in solution (*c*) to the reference state $r`$. To implement this approach, single *reference states* common to (a) *all* free ligands, (b) *all* free proteins, and (c) *all* protein-ligand complexes must be generated. Two simulations are required to calculate the free

energy change along the alchemical reaction converting the WT protein to the reference state in the bound and unbound form. Each $\Delta\Delta G$ estimate requires two subsequent simulations to convert the mutant protein to the reference state in the bound and unbound form.

j. Single reference single mutant (SRSM) approach

An alternative approach can be carried out by considering the thermodynamic cycle depicted in Fig. S2B. Given an inhibitor *I*, such an approach allows for the computation of $\Delta\Delta G$ between two proteins (e.g., the WT enzyme *W* and a specific mutant *M*) as:

$$\Delta\Delta G^{W\to M}(I) = \Delta G_{fc}^{M-I} - \Delta G_{fc}^{W-I} = \Delta G_{fr}^{M} - \Delta G_{fr}^{W} + \Delta G_{cr'}^{W-I} - \Delta G_{cr'}^{M-I}. \qquad (5)$$

Implementation of this approach requires the construction of common reference states for (a) the free WT enzyme and the mutant and (b) the complexes of the WT enzyme and the mutant with the inhibitor. Since the inhibitor remains unaltered, we note that in this case the *reference state* for the free inhibitor and the state of the free inhibitor in solution are identical (i.e., the term ΔG_{fr}^{I} in Fig. S2B is null). Consequently, four simulations were required to compute a single estimate of $\Delta\Delta G^{W\to M}(I)$, two involving the alchemic reactions of converting the free WT enzyme and the free mutant to the common reference state *r* and two for converting the complexes of (a) the inhibitor bound to the WT and (b) mutant to a common state *r`*.

k. Estimation of the binding affinity using the Molecular Mechanics – Poisson Boltzmann Surface Area / Generalized Born Surface Area (MM-PBSA/GBSA) method

A utility PERL script provided with the LEAD software was used to merge the data associated with the different replicas from a given HREX-SRTI run into a single trajectory consisting of snapshots taken every 1 ps. Trajectories from the different runs were used in subsequent

analyses. The MM-PBSA/GBSA method [26], as implemented in Amber10, was used to obtain additional estimates of the changes in binding free energy. All trajectories from the runs corresponding to complexes of oseltamivir and zanamivir with the WT and mutant NA molecules were used. The MM-PBSA/GBSA calculations were carried out by including all frames from our SRSM trajectories.

The binding free energy ΔG^{M-I} associated with the formation of the complex of protein *M* and inhibitor *I* was computed according to the following equation:

$$\Delta G^{M-I} = G_c^{M-I} - G_f^{M} - G_f^{I} \qquad (6)$$

where G_c^{M-I}, G_f^{M}, and G_f^{I} are the free energies of the complex, the protein free in solution and the ligand free in solution, respectively. Following the MM/PBSA approach, the free energy of a given component *x* (i.e., protein, inhibitor, or protein-inhibitor complex) was computed as:

$$G^{x} = H_{gas}^{x} + G_{solv}^{x} - TS \qquad (7)$$

where H_{gas}^{x} was computed as the energy of component *x* in the gas phase, *TS* is the entropic contribution of *x* at temperature *T*, and G_{solv}^{x} represents the solvation free energy of *x* that includes polar and non-polar solvation energy. It was computed as:

$$G_{solv}^{x} = G_{surf}^{x} + G_{PB}^{x} \qquad (8)$$

with $G_{surf}^{x} = \gamma A + b$ being the nonpolar solvation term, where *A* is the solvent accessible surface of component *x*, and $\gamma = 0.00542$ and $b = 0.92$ are constants, and the term G_{PB}^{x} accounts for the

electrostatic interaction between solute and solvent. Evaluation of the entropic contribution related to equation (7), $T\Delta S$, was carried out with the NMODE module from Amber10 using snapshots taken from each trajectory every 500 ps.

l. Estimation of the binding affinity using RosettaInterface

RosettaInterface [27] uses computational mutagenesis to predict the change in binding free energy of a protein-protein interaction associated with a point mutation of an interface residue. This algorithm has been used in a wide range of applications, including hotspot detection [27], predictive docking [28], and validation of predicted protein interactions [29]. We adapted the RosettaInterface algorithm that was designed for protein-protein interactions to predict binding free energy changes in protein-ligand interactions. We implemented the algorithm using a PyRosetta [30] script in the Rosetta v3.3 software package [31]. We kept the RosettaInterface algorithm essentially unchanged but used the parameterization and score functions from RosettaLigand [32] to model protein-ligand interactions.

In RosettaInterface, the same refinement protocol was applied to both the wild type (W) and mutant (M) complexes, after which the binding energy was calculated for each complex. Prior to refinement, the M complex was created by applying the necessary mutation to the W complex using Rosetta's Monte Carlo-simulated annealing side-chain packer [33]. During refinement the protein-ligand interfaces were optimized using the side-chain packer under the RosettaLigand *ligand_soft_rep* score function. The resulting refined complexes serve as 'bound' structures for the binding energy calculation. Unbound structures were generated starting from the bound structure and translating the ligand 500 Å away. The change in binding free energy, $\Delta\Delta G^{W\rightarrow M}(I)$, is then calculated using the same score function used in the refinement:

$$\Delta\Delta G^{W \to M}(I) = (\Delta G_c^{M-I} - \Delta G_f^{M}) - (\Delta G_c^{W-I} - \Delta G_f^{W}) \tag{9}$$

where ΔG_c^{M-I} and ΔG_c^{W-I} are the free energy changes due to the formation of the complexes of the mutant *M* with inhibitor *I*, and the *W* enzyme with inhibitor *I*, respectively, and ΔG_f^{M} and ΔG_f^{W} are the free energy changes associated with the formation of the free mutant and WT enzymes, respectively.

Two starting structures were used based on the crystal structures of N1 NA N294S bound to oseltamivir and N1 NA H274Y bound to zanamivir [17]. Oseltamivir and zanamivir were parameterized with default settings using the *molfile_to_params.py* script in Rosetta v3.3. The side-chain packer used an expanded rotamer library that also included additional rotamers representing the initial crystallographic side-chain conformations [34]. We carried out the RosettaInterface algorithm ten independent times and both the means and standard deviations of $\Delta\Delta G^{W \to M}(I)$ are listed in Table 1. RosettaInterface calculated the WT binding energy as -11.6 kcal/mol and -12.7 kcal/mol for oseltamivir and zanamivir, respectively. The predicted $\Delta\Delta G$ of the mutants ranged from -0.9 kcal/mol to +1.0 kcal/mol. A weak correlation was observed between the predicted and experimentally determined $\Delta\Delta G$ for oseltamivir ($R^2 = 0.77$) and no correlation was observed for zanamivir ($R^2 = 0.05$). The lack of significant correlation is unsurprising because RosettaInterface was primarily intended to classify mutations as stabilizing or destabilizing, and not to quantitatively predict the experimental $\Delta\Delta G$.

2. Key NA-inhibitor interactions

Zanamivir and oseltamivir are both sialic acid derivatives and share many common interactions with NA (see Fig. S3). However, there are also several differences, some of which have not been

previously observed in the crystal structures [17]. A triad of positively charged residues, R118, R292, and R371, played a major role in zanamivir binding, forming salt bridges with the carboxyl group at C1. In contrast, the C1 carboxyl group of oseltamivir primarily interacted with R292 and R371, with R118 contributing little to the binding energy. A group of negatively charged residues, D151, E227, and E119, interacted with the C4 ammonium group of oseltamivir, forming particularly strong interactions with E119. In zanamivir, the corresponding guanidinium group interacted primarily with E119 and E227 but not D151. The charged residues R224, E276, and E277 formed a network of hydrogen bonds with the polar tail of zanamivir, while the nonpolar tail of oseltamivir formed only weakly stabilizing interactions with E277 and the nearby I222. Finally, there was an extensive network of intramolecular hydrogen bonds and salt bridges among binding site residues that could play a significant role in mediating drug resistance.

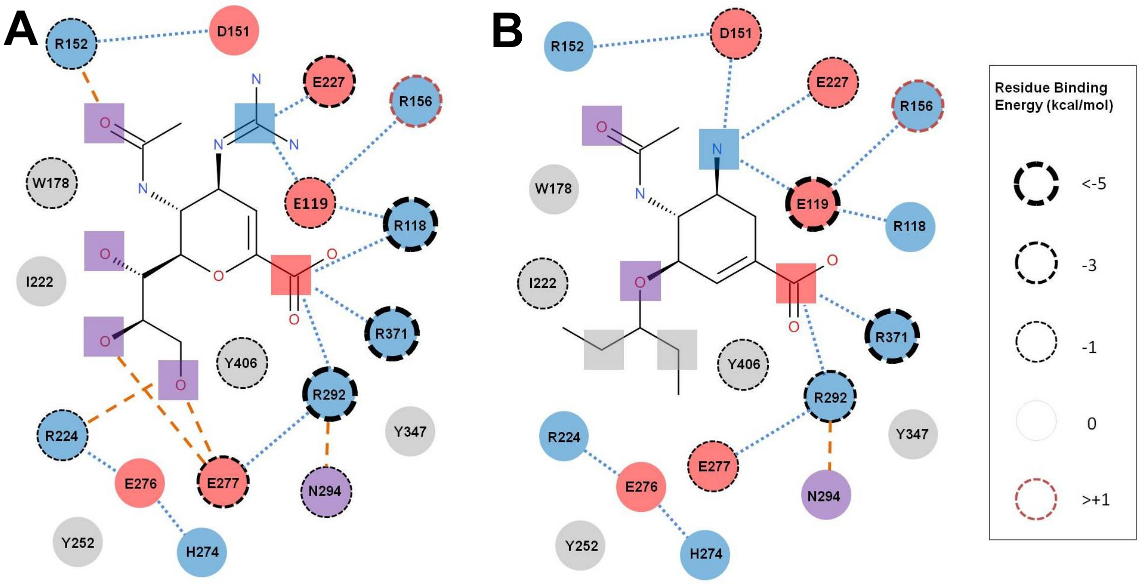

Figure S3: Interaction map of NA with zanamivir (**A**) and oseltamivir (**B**) based on MM-GBSA binding energy decomposition of composite WT SRSM/HREX trajectories. Residues are colored by type: red (negatively charged), blue (positively charged), purple (polar), and gray (nonpolar).

Salt bridges are denoted by blue dotted lines, hydrogen bonds by orange dotted lines. The contributions of each residue to the MM-GBSA binding energy is depicted with contours of different thickness as specified in the legend. Select chemical groups on the inhibitor are noted as squares with the same color theme as the protein residues.

3. The H274Y mutant: Constraint imposed on the χ_1 dihedral angle of E276 upon mutation

Figure S4 shows the distribution of the χ_1 dihedral angle of E276 for runs involving the WT and the H274Y mutant. Two peaks around $\chi_1 \sim 60^o$ and $\chi_1 \sim -60^o$ were observed in the distribution for free WT (red line), with *gauche+* and *gauche–* conformations evenly visited. In complexes of the WT with both oseltamivir and zanamivir, the *gauche+* conformation around χ_1 is generally preferred. A third small peak is present for $\chi_1 \sim -160^o$ in runs of the WT enzyme with oseltamivir. The χ_1 value observed in the experimental structure [17] 2HTY for the free WT is -89°. The introduction of the H274Y mutation leads to a dramatic shift in the accessible space of the E276 side chain. As shown in Fig. S4 (light-blue and orange lines), the χ_1 dihedral angle distributions are practically flat around $\chi_1 = -60^o$ for both oseltamivir and zanamivir, indicating that such a region is highly restricted to the GLU side chain in the mutant. The value of χ_1 derived from the experimental structure of the complex of the H247Y mutant with oseltamivir (PDB code: 3CL0) [17] is -157°, while that obtained from the experimental structure of the H247Y mutant in complex with zanamivir (PDB code: 3CKZ) [17] is -167°.

The *trans* conformation ($\sim 180^o$) of the dihedral angle χ_1 of E276 showed increase sampling in runs involving the H274Y mutant as compared to those corresponding to complexes of the WT enzyme. However, we observed that the *gauche+* conformation, which was not detected experimentally, was the most dominant one in our simulations. While it is possible that the

disagreement is due to inaccuracy in the forcefield parameters, visual inspection of the superposition of a few conformations from the molecular dynamics trajectories onto the X-ray structures 2HTY and 3CKZ shows (Figs. S5A-B) that the side chain of E276 in *gauche*+ conformation can fit quite well in the electron density maps of the two experimental structures. In either the *gauche*+ or *trans* conformation, the carboxyl group of E176 is found displaced toward the center of the binding site in the H274Y mutant.

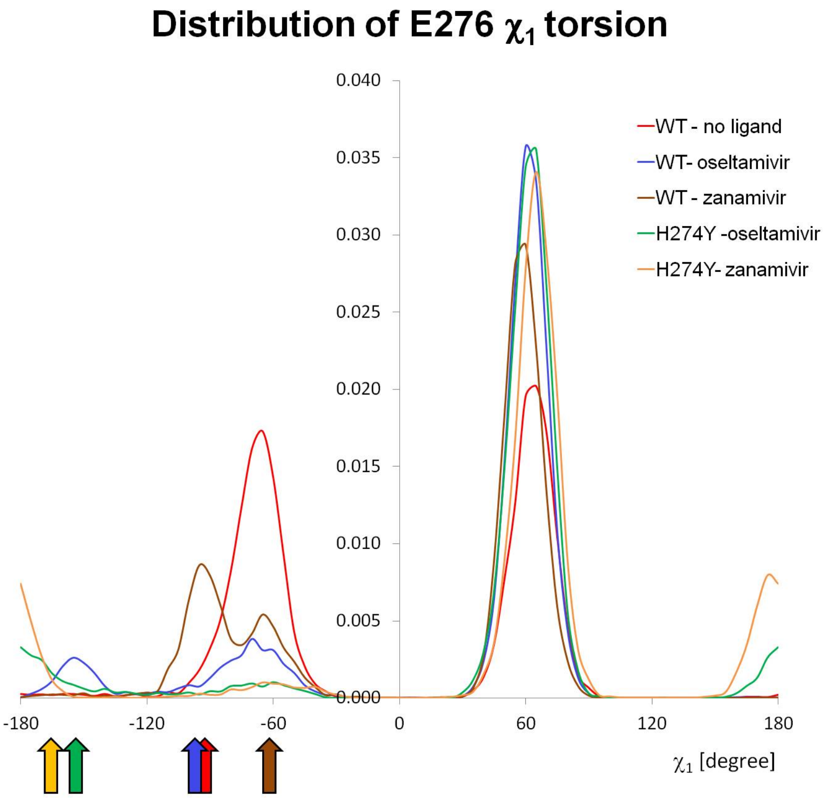

Figure S4: Distribution of the χ_1 dihedral angle of residue E276 for the WT free enzyme, and the WT and H274Y mutant enzymes in complexes with oseltamivir and zanamivir derived from runs produced with the *SRSM* approach. The χ_1 values observed in the experimental structures are (a) WT NA free enzyme: -89° (2HTY) [17] (the red arrow indicates the approximate position of the

experimental value); (b) WT NA in complexes with oseltamivir [35]: -86° (2HU0) and -108° (2HU4) (the blue arrow indicates the approximate position of the average experimental value); (c) WT NA in complexes with zanamivir: -61° (3B7E) [36] indicated with a brown arrow; (d) the H274Y mutant in complex with oseltamivir: -157° (3CL0) [17] indicated with a green arrow; and (e) the H274Y mutant in complex with zanamivir: -167° (3CKZ) [17] indicated with an orange arrow.

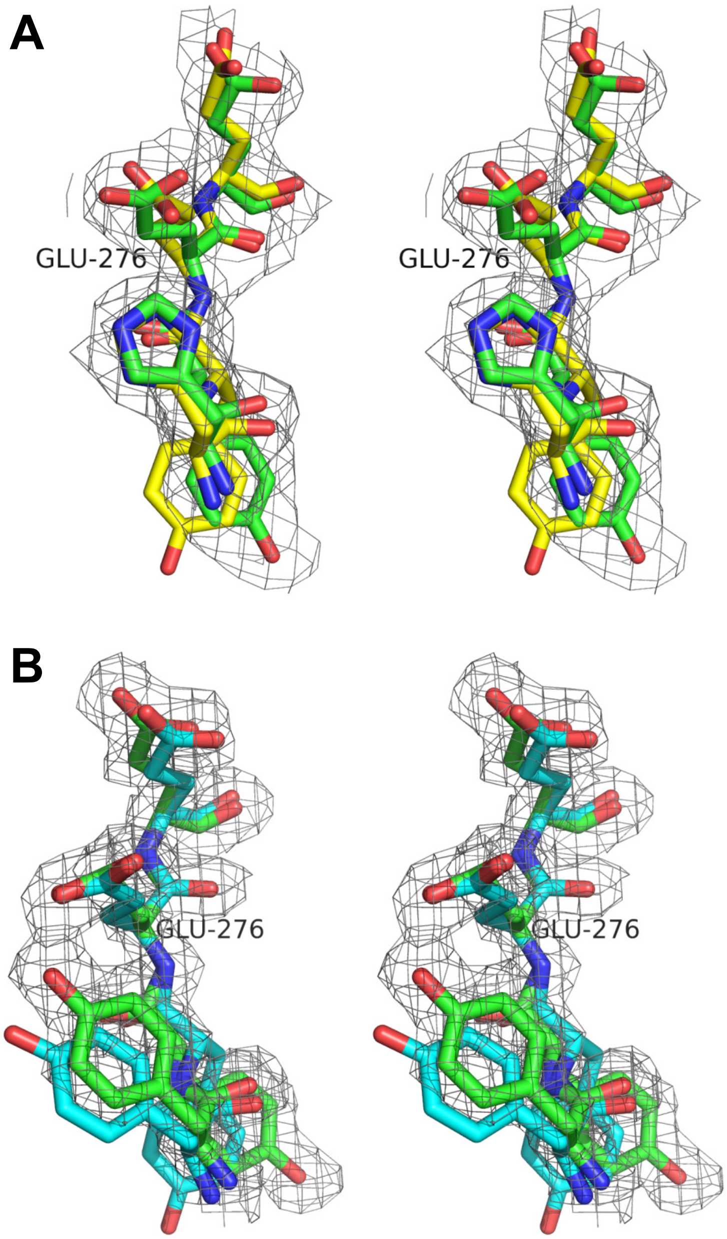

Figure S5: Comparison of E276 conformations from simulations and experiments. (**A**) Stereo view of the superposition of residues 274–277 of NA from the experimental structure 2HTY (carbon atoms colored green) and a snapshot (carbon atoms shown in yellow) from one of the SRSM trajectories. The snapshot shows the side chain of E276 in the *gauche+* conformation, which is the most preferred according to the simulations. Both experimental and calculated conformers of the E276 side chain fit well in the electron density map of 2HTY. (**B**) Stereo view

of the superposition of residues 274–277 of NA from the experimental structure 3CKZ (carbon atoms colored green) and a snapshot (carbon atoms shown in cyan) from one of the SRSM trajectories. The snapshot shows the side chain of E276 in the *gauche+* conformation, which is the most preferred according to the simulations. Both experimental and calculated conformers of the E276 side chain fit well in the electron density map of 3CKZ.

4. The N294S mutation: The Y347 side chain

Figure 5C-D shows the distribution of distances between the Y347 O_η atom and N_η atoms of R292 and R371 obtained from our SRSM simulations. The Y347 O_η atom in the WT enzyme tended to interact persistently with the carboxyl group of the inhibitors, thus remaining in close proximity to R292 and R371 (Fig. 5C). The N294S mutation modified the conformational preference of the backbone of Y347 by bringing its C=O group toward R292. The new backbone conformation of Y347 in turns affected the dynamics of the TYR side chain, as shown by the different distance patterns presented in Fig. 5D. However, it must be noted that the conformations accessible to Y347 in the N294S mutant seem different from those reported earlier [37]. We observed that the "out" conformations described by Lawrenz et al. [37] were rarely accessed in our simulations. Instead, conformations in which the phenol group and the carboxyl group of the ligands did not form H-bonds tended to keep the aromatic tyrosine ring near the binding pocket and inhibitors.

Finally, it is worth noting that the effect of the Y347 side chain on resistance is suspect. Indeed, certain strains of N1 have asparagine in place of tyrosine. Among the most notable strains are the recombinant viruses associated with the Spanish flu of 1918 that were found to be sensitive in

both tissue culture and mice to oseltamivir [38]. In those NAs, the side chain of the N347 residue does not interact directly with the carboxyl group of the inhibitor (e.g., PDB: 3B7E) [36].

5. References

1. Wang J, Wolf RM, Caldwell JW, Kollman PA, Case DA (2004) Development and testing of a general amber force field. J Comput Chem 25: 1157–1174.
2. Khavrutskii IV, Wallqvist A (2010) Computing relative free energies of solvation using single reference thermodynamic integration augmented with hamiltonian replica exchange. J Chem Theory Comput 6: 3427–3441.
3. Khavrutskii IV, Wallqvist A (2011) Improved binding free energy predictions from single-reference thermodynamic integration augmented with hamiltonian replica exchange. J Chem Theory Comput 7: 3001–3011.
4. Weininger D (1988) SMILES, a chemical language and information system. 1. Introduction to methodology and encoding rules. J Chem Inf Comput Sci 28: 31–36.
5. Weininger D, Weininger A, Weininger JL (1989) SMILES. 2. Algorithm for generation of unique SMILES notation. J Chem Inf Comp Sci 29: 97–101.
6. OpenEye Scientific Software I (2010) OEChem. version 1.7.4 ed. Santa Fe, NM, USA: OpenEye Scientific Software, Inc.
7. Boström J, Greenwood JR, Gottfries J (2003) Assessing the performance of OMEGA with respect to retrieving bioactive conformations. J Mol Graphics and Mod 21: 449–462.
8. Dewar MJS, Zoebisch EG, Healy EF, Stewart JJP (1985) AM1: A new general purpose quantum mechanical molecular model. J Am Chem Soc 107: 3902–3909.
9. Stewart JJP (2006) MOPAC7. http://sourceforge.net/projects/mopac7/ (accessed on May 15, 2012).
10. Jakalian A, Bush BL, Jack DB, Bayly CI (2000) Fast, efficient generation of high-quality atomic charges. AM1-BCC model: I. Method. J Comput Chem 21: 132–146.
11. Wang J (2009) Antechamber. http://ambermd.org/ (accessed on May 15, 2012).
12. Wang J, Wang W, Kollman PA, Case DA (2006) Automatic atom type and bond type perception in molecular mechanical calculations. J Mol Graph Model 25: 247–260.
13. Nicholls A, Mobley DL, Guthrie JP, Chodera JD, Bayly CI, et al. (2008) Predicting small-molecule solvation free energies: an informal blind test for computational chemistry. J Med Chem 51: 769–779.
14. Mobley DL, Bayly CI, Cooper MD, Shirts MR, Dill KA (2009) Small molecule hydration free energies in explicit solvent: An extensive test of fixed-charge atomistic simulations. J Chem Theory Comput 5: 350−358.
15. Mobley DL, Bayly CI, Cooper MD, Dill KA (2009) Predictions of hydration free energies from all-atom molecular dynamics simulations. J Phys Chem B 113: 4533–4537.
16. Shivakumar D, Deng Y, Roux B (2009) Computations of absolute solvation free energies of small molecules using explicit and implicit solvent model. J Chem Theory Comput 5: 919–930.
17. Collins PJ, Haire LF, Lin YP, Liu J, Russell RJ, et al. (2008) Crystal structures of oseltamivir-resistant influenza virus neuraminidase mutants. Nature 453: 1258–1261.
18. Word JM, Lovell SC, Richardson JS, Richardson DC (1999) Asparagine and glutamine: using hydrogen atom contacts in the choice of sidechain amide orientation. J Mol Biol 285: 1735–1747.
19. Case DA, Darden TA, Cheatham I, T.E. , Simmerling CL, Wang J, et al. (2008) AMBER 10. San Francisco: University of California.
20. Berendsen HJC, van dSD, van DR (1995) GROMACS: A message-passing parallel molecular dynamics implementation. Comput Phys Commun 91: 43–56.

21. Hess B, Kutzner C, van dSD, Lindahl E (2008) GROMACS 4: Algorithms for highly efficient, load-balanced, and scalable molecular simulation. J Chem Theory Comput 4: 435–447.
22. Van Der Spoel D, Lindahl E, Hess B, Groenhof G, Mark AE, et al. (2005) GROMACS: fast, flexible, and free. J Comput Chem 26: 1701–1718.
23. van der Spoel D, Lindahl E, Hess B, Kutzner C, van Buuren AR, et al. (2006) GROMACS User Manual Version 4.0. 4.0 ed. AG Groningen, The Netherlands: The GROMACS development team.
24. Hess B, Bekker H, Berendsen HJC, Fraaije JGEM (1997) LINCS: A linear constraint solver for molecular simulations. J Comput Chem 18: 1463–1472.
25. Cardellina II JH, Roxas-Duncan VI, Montgomery V, Eccard V, Campbell Y, et al. (2012) Fungal bis-naphthopyrones as inhibitors of botulinum neurotoxin serotype A. ACS Med Chem Lett: [Online early access]. DOI: 10.1021/ml200312s. Published Online: April 200312, 202012.
26. Srinivasan J, Cheatham I, T.E., , Cieplak P, Kollman PA, Case DA (1998) Continuum solvent studies of the stability of DNA, RNA and phosphoramidate-DNA helices. J Am Chem Soc 120: 9401–9409.
27. Kortemme T, Baker D (2002) A simple physical model for binding energy hot spots in protein-protein complexes. Proc Natl Acad Sci USA 99: 14116–14121.
28. Chaudhury S, Sircar A, Sivasubramanian A, Berrondo M, Gray JJ (2007) Incorporating biochemical information and backbone flexibility in RosettaDock for CAPRI rounds 6-12. Proteins 69: 793–800.
29. Sivasubramanian A, Chao G, Pressler HM, Wittrup KD, Gray JJ (2006) Structural model of the mAb 806-EGFR complex using computational docking followed by computational and experimental mutagenesis. Structure 14: 401–414.
30. Chaudhury S, Lyskov S, Gray JJ (2011) PyRosetta: a script-based interface for implementing molecular modeling algorithms using Rosetta. Bioinformatics 26: 689–691.
31. Leaver-Fay A, Tyka M, Lewis SM, Lange OF, Thompson J, et al. (2011) ROSETTA3: an object-oriented software suite for the simulation and design of macromolecules. Methods Enzymol 487: 545–574.
32. Davis IW, Baker D (2009) RosettaLigand docking with full ligand and receptor flexibility. J Mol Biol 385: 381–392.
33. Kuhlman B, Baker D (2000) Native protein sequences are close to optimal for their structures. Proc Natl Acad Sci USA 97: 10383–10388.
34. Wang C, Schueler-Furman O, Baker D (2005) Improved side-chain modeling for protein-protein docking. Protein Sci 14: 1328–1339.
35. Russell RJ, Haire LF, Stevens DJ, Collins PJ, Lin YP, et al. (2006) The structure of H5N1 avian influenza neuraminidase suggests new opportunities for drug design. Nature 443: 45–49.
36. Xu X, Zhu X, Dwek RA, Stevens J, Wilson IA (2008) Structural characterization of the 1918 influenza virus H1N1 neuraminidase. J Virol 82: 10493–10501.
37. Lawrenz M, Wereszczynski J, Amaro R, Walker R, Roitberg A, et al. (2010) Impact of calcium on N1 influenza neuraminidase dynamics and binding free energy. Proteins 78: 2523–2532.

38. Tumpey TM, García-Sastre A, Mikulasova A, Taubenberger JK, Swayne DE, et al. (2002) Existing antivirals are effective against influenza viruses with genes from the 1918 pandemic virus. Proc Natl Acad Sci USA 99: 13849–13854.
39. Massova I, Kollman PA (2000) Combined molecular mechanical and continuum solvent approach (MM-PBSA/GBSA) to predict ligand binding. Perspec Drug Discov 18: 113–135.
40. Eisenhaber F, Lijnzaad P, Argos P, Sander C, Scharf M (1995) The double cube lattice method: efficient approaches to numerical integration of surface area and volume and to dot surface contouring of molecular assemblies. J Comp Chem 16: 273–284.

Appendix 1: mol2 file for oseltamivir

```
@<TRIPOS>MOLECULE
UNK
   44    44     1     0     0
SMALL
No Charge or Current Charge


@<TRIPOS>ATOM
      1 C1          0.8350    0.9420    1.0980 c2         1 UNK     -0.239593
      2 C2          2.1730    0.9420    1.0980 ce         1 UNK     -0.164712
      3 C3          2.9900    2.2210    1.0980 c          1 UNK      0.915790
      4 C4          1.1580   -3.6120   -0.4620 c          1 UNK      0.661732
      5 C5          2.9230   -0.2400    0.5820 c3         1 UNK     -0.061927
      6 C6          0.0020   -0.1350    0.4780 c3         1 UNK      0.162673
      7 C7          0.7970   -1.1810   -0.3690 c3         1 UNK      0.094078
      8 C8          2.2200   -0.6300   -0.7280 c3         1 UNK      0.105106
      9 C9          1.1930   -4.9200    0.2830 c3         1 UNK     -0.172969
     10 C10        -4.2850    0.3640    0.9530 c3         1 UNK     -0.094801
     11 C11        -1.4950    3.4260   -0.5720 c3         1 UNK     -0.094801
     12 C12        -3.1610    0.0060    0.0170 c3         1 UNK     -0.094677
     13 C13        -2.3810    2.2450   -0.8680 c3         1 UNK     -0.094677
     14 C14        -2.0520    1.0550    0.0310 c3         1 UNK      0.143542
     15 N1          0.8660   -2.4680    0.2670 n          1 UNK     -0.573838
     16 N2          2.1240    0.5750   -1.6140 n4         1 UNK     -0.893692
     17 O1          3.3650    2.5250   -0.0990 o          1 UNK     -0.772985
     18 O2          3.2450    2.8830    2.1150 o          1 UNK     -0.772985
     19 O3          1.3650   -3.5380   -1.6900 o          1 UNK     -0.611770
     20 O4         -0.8540    0.4720   -0.5050 os         1 UNK     -0.456529
     21 H1          0.2680    1.8150    1.4620 ha         1 UNK      0.155805
     22 H2          4.0000    0.0180    0.3940 hc         1 UNK      0.082387
     23 H3          2.9010   -1.0920    1.3060 hc         1 UNK      0.082387
     24 H4         -0.6240   -0.6480    1.2610 h1         1 UNK      0.065115
     25 H5          0.2350   -1.3300   -1.3460 h1         1 UNK      0.095400
     26 H6          2.7970   -1.4250   -1.2800 hx         1 UNK      0.103468
     27 H7          0.1970   -5.1340    0.7410 hc         1 UNK      0.073307
     28 H8          1.4440   -5.7360   -0.4380 hc         1 UNK      0.073307
     29 H9          1.9670   -4.8950    1.0880 hc         1 UNK      0.073307
     30 H10        -5.0780   -0.4220    0.9180 hc         1 UNK      0.038480
     31 H11        -3.9170    0.4480    2.0040 hc         1 UNK      0.038480
     32 H12        -4.7470    1.3400    0.6660 hc         1 UNK      0.038480
     33 H13        -1.6890    4.2500   -1.3000 hc         1 UNK      0.038480
     34 H14        -1.6820    3.8160    0.4590 hc         1 UNK      0.038480
     35 H15        -0.4180    3.1330   -0.6410 hc         1 UNK      0.038480
     36 H16        -2.7240   -0.9850    0.3140 hc         1 UNK      0.051378
     37 H17        -3.5500   -0.1060   -1.0280 hc         1 UNK      0.051378
     38 H18        -2.2620    1.9350   -1.9390 hc         1 UNK      0.051378
     39 H19        -3.4560    2.5200   -0.7080 hc         1 UNK      0.051378
     40 H20        -1.8710    1.4090    1.0840 h1         1 UNK      0.054473
     41 H21         0.8560   -2.5020    1.2580 hn         1 UNK      0.322402
     42 H22         2.4240    0.3480   -2.5540 hn         1 UNK      0.466429
     43 H23         2.7390    1.3850   -1.1960 hn         1 UNK      0.466429
     44 H24         1.1590    0.9130   -1.6330 hn         1 UNK      0.466429
@<TRIPOS>BOND
```

```
     1     1     2 2
     2     1     6 1
     3     2     3 1
     4     2     5 1
     5     3    17 1
     6     3    18 1
     7     4     9 1
     8     4    15 1
     9     4    19 2
    10     5     8 1
    11     6     7 1
    12     6    20 1
    13     7     8 1
    14     7    15 1
    15     8    16 1
    16    10    12 1
    17    11    13 1
    18    12    14 1
    19    13    14 1
    20    14    20 1
    21     1    21 1
    22     5    22 1
    23     5    23 1
    24     6    24 1
    25     7    25 1
    26     8    26 1
    27     9    27 1
    28     9    28 1
    29     9    29 1
    30    10    30 1
    31    10    31 1
    32    10    32 1
    33    11    33 1
    34    11    34 1
    35    11    35 1
    36    12    36 1
    37    12    37 1
    38    13    38 1
    39    13    39 1
    40    14    40 1
    41    15    41 1
    42    16    42 1
    43    16    43 1
    44    16    44 1
@<TRIPOS>SUBSTRUCTURE
     1 UNK         1 TEMP              0 ****  ****    0 ROOT
```

Appendix 2: mol2 file for zanamivir

```
@<TRIPOS>MOLECULE
UNK
   43    43     1     0     0
SMALL
No Charge or Current Charge


@<TRIPOS>ATOM
      1 C1         -0.8960    1.9140   -1.0400 c2         1 UNK     -0.406952
      2 C2          0.4530    1.9140   -1.0400 ce         1 UNK      0.067843
      3 C3          1.3270    3.1900   -1.0400 c          1 UNK      0.940133
      4 C4         -2.1850   -2.0120    1.2060 c          1 UNK      0.680510
      5 C5         -1.9100    1.9850    1.7390 cz         1 UNK      0.484163
      6 C6         -1.6920    0.8060   -0.4560 c3         1 UNK      0.074197
      7 C7         -0.7640   -0.3860   -0.0010 c3         1 UNK      0.040044
      8 C8          0.4660   -0.4500   -0.9340 c3         1 UNK      0.092881
      9 C9         -2.6760   -3.4340    1.2840 c3         1 UNK     -0.176203
     10 C10         3.2300   -0.4420   -2.1260 c3         1 UNK      0.112979
     11 C11         1.4940   -1.5210   -0.5270 c3         1 UNK      0.126826
     12 C12         2.5640   -1.7280   -1.6190 c3         1 UNK      0.095073
     13 N13        -1.1110    3.0650    1.5350 nh         1 UNK     -0.467873
     14 N14        -2.2750    1.6450    3.0320 nh         1 UNK     -0.528014
     15 N15        -1.4890   -1.6310    0.0770 n          1 UNK     -0.559251
     16 N16        -2.5120    1.2820    0.6670 nh         1 UNK     -0.333106
     17 O17         0.8290    4.1170   -0.3430 o          1 UNK     -0.774520
     18 O18         2.4180    3.1600   -1.6420 o          1 UNK     -0.774520
     19 O19        -2.4300   -1.1870    2.1180 o          1 UNK     -0.681643
     20 O20         1.1900    0.7850   -0.8200 os         1 UNK     -0.306835
     21 O21         3.7210    0.3090   -1.0340 oh         1 UNK     -0.633136
     22 O22         2.0810   -1.2050    0.7170 oh         1 UNK     -0.606048
     23 O23         3.5350   -2.6340   -1.1310 oh         1 UNK     -0.595939
     24 H1         -1.4480    2.7980   -1.3930 ha         1 UNK      0.170637
     25 H6         -2.4480    0.4350   -1.2140 h1         1 UNK      0.089011
     26 H7         -0.3580   -0.1490    1.0340 h1         1 UNK      0.085264
     27 H8          0.1450   -0.5880   -2.0010 h1         1 UNK      0.069342
     28 H91        -1.8070   -4.1330    1.3610 hc         1 UNK      0.076184
     29 H92        -3.3130   -3.5470    2.1950 hc         1 UNK      0.076184
     30 H93        -3.2800   -3.7000    0.3830 hc         1 UNK      0.076184
     31 H10         2.5000    0.1480   -2.7380 h1         1 UNK      0.053724
     32 H3          4.1140   -0.7270   -2.7610 h1         1 UNK      0.053724
     33 H11         0.9810   -2.5080   -0.3500 h1         1 UNK      0.071787
     34 H12         2.0960   -2.2600   -2.4950 h1         1 UNK      0.069070
     35 H13        -0.7060    3.5430    2.3090 hn         1 UNK      0.344520
     36 H4         -0.7680    3.3450    0.6160 hn         1 UNK      0.344520
     37 H14        -1.9590    2.1760    3.8070 hn         1 UNK      0.313395
     38 H5         -2.6980    0.7590    3.2070 hn         1 UNK      0.313395
     39 H15        -1.2850   -2.3180   -0.6110 hn         1 UNK      0.321056
     40 H16        -3.1460    0.5630    0.9790 hn         1 UNK      0.280645
     41 H21         3.3860    1.2190   -1.1280 ho         1 UNK      0.455524
     42 H22         2.6210   -0.4070    0.5940 ho         1 UNK      0.440732
     43 H23         3.9880   -2.1880   -0.4010 ho         1 UNK      0.424498
@<TRIPOS>BOND
     1    1    2 2
     2    1    6 1
     3    1   24 1
     4    2    3 1
     5    2   20 1
     6    3   17 1
     7    3   18 1
     8    4    9 1
     9    4   15 1
```

```
    10    4   19 2
    11    5   13 2
    12    5   14 1
    13    5   16 1
    14    6    7 1
    15    6   16 1
    16    6   25 1
    17    7    8 1
    18    7   15 1
    19    7   26 1
    20    8   11 1
    21    8   20 1
    22    8   27 1
    23    9   28 1
    24    9   29 1
    25    9   30 1
    26   10   12 1
    27   10   21 1
    28   10   31 1
    29   10   32 1
    30   11   12 1
    31   11   22 1
    32   11   33 1
    33   12   23 1
    34   12   34 1
    35   13   35 1
    36   13   36 1
    37   14   37 1
    38   14   38 1
    39   15   39 1
    40   16   40 1
    41   21   41 1
    42   22   42 1
    43   23   43 1
@<TRIPOS>SUBSTRUCTURE
     1 UNK         1 TEMP              0 ****  ****    0 ROOT
```

Appendix 3: mol2 file for core-ligand

```
@<TRIPOS>MOLECULE
UNK
   21    21     1     0     0
SMALL
No Charge or Current Charge


@<TRIPOS>ATOM
      1 C1         -1.4980   -1.1110    0.0040 ca        1 UNK     -0.089740
      2 C2          0.9060   -1.1110    0.0040 ca        1 UNK     -0.089740
      3 C3         -1.5080    0.2780    0.0040 ca        1 UNK     -0.189440
      4 C4          0.9230    0.2830    0.0040 ca        1 UNK     -0.189440
      5 C5         -0.2940   -1.8230    0.0040 ca        1 UNK     -0.137990
      6 C6         -0.2890    0.9990    0.0040 ca        1 UNK      0.019410
      7 C7         -0.3020   -3.3430    0.0040 c         1 UNK      0.909010
      8 C8          0.6770    3.3190   -0.0090 c         1 UNK      0.655810
      9 C9          0.2690    4.7820   -0.0080 c3        1 UNK     -0.180390
     10 N10        -0.3580    2.4100    0.0050 n         1 UNK     -0.459990
     11 O11         0.8100   -3.9430    0.0050 o         1 UNK     -0.832240
     12 O12        -1.4240   -3.9270    0.0040 o         1 UNK     -0.832240
     13 O13         1.8860    3.0150   -0.0200 o         1 UNK     -0.609090
     14 H1         -2.4410   -1.6830    0.0040 ha        1 UNK      0.153510
     15 H2          1.8500   -1.6800    0.0040 ha        1 UNK      0.153510
     16 H3         -2.4710    0.8090    0.0040 ha        1 UNK      0.125010
     17 H4          1.8870    0.8140    0.0030 ha        1 UNK      0.125010
     18 H91        -0.3220    5.0240    0.9070 hc        1 UNK      0.055943
     19 H92        -0.3410    5.0190   -0.9120 hc        1 UNK      0.055943
     20 H93         1.1930    5.4070   -0.0190 hc        1 UNK      0.055943
     21 H10        -1.2800    2.7820    0.0110 hn        1 UNK      0.301210
@<TRIPOS>BOND
     1    1    3 ar
     2    1    5 ar
     3    1   14 1
     4    2    4 ar
     5    2    5 ar
     6    2   15 1
     7    3    6 ar
     8    3   16 1
     9    4    6 ar
    10    4   17 1
    11    5    7 1
    12    6   10 1
    13    7   11 1
    14    7   12 1
    15    8    9 1
    16    8   10 1
    17    8   13 2
    18    9   18 1
    19    9   19 1
    20    9   20 1
    21   10   21 1
@<TRIPOS>SUBSTRUCTURE
     1 UNK         1 TEMP              0 ****  ****    0 ROOT
```

Appendix 4: mapping files for SRMM calculations

NOTE: Since 80 N-terminal residues are missing in the experimental structures, residues 252, 274 and 294 in the original NA sequences are labeled as 172, 194, 214, respectively in the following files.

<u>*WT to core*</u>

```
172:TYR:C->     172:ASN:C
172:TYR:O->     172:ASN:O
172:TYR:N->     172:ASN:N
172:TYR:H->     172:ASN:H
172:TYR:CA->    172:ASN:CA
172:TYR:HA->    172:ASN:HA
172:TYR:CB->    172:ASN:CB
172:TYR:2HB->   172:ASN:2HB
172:TYR:3HB->   172:ASN:3HB
172:TYR:CG->    172:ASN:CG
172:TYR:CD1->   172:ASN:OD1
172:TYR:CD2->   172:ASN:ND2
194:HIP:C->     194:ASN:C
194:HIP:O->     194:ASN:O
194:HIP:N->     194:ASN:N
194:HIP:H->     194:ASN:H
194:HIP:CA->    194:ASN:CA
194:HIP:HA->    194:ASN:HA
194:HIP:CB->    194:ASN:CB
194:HIP:2HB->   194:ASN:2HB
194:HIP:3HB->   194:ASN:3HB
194:HIP:CG->    194:ASN:CG
194:HIP:ND1->   194:ASN:OD1
194:HIP:CD2->   194:ASN:ND2
214:ASN:C->     214:SER:C
214:ASN:O->     214:SER:O
214:ASN:N->     214:SER:N
214:ASN:H->     214:SER:H
214:ASN:CA->    214:SER:CA
214:ASN:HA->    214:SER:HA
214:ASN:CB->    214:SER:CB
214:ASN:2HB->   214:SER:2HB
214:ASN:3HB->   214:SER:3HB
214:ASN:CG->    214:SER:OG
```

<u>*mutant H274Y to core*</u>

```
172:TYR:N->  172:ASN:N
172:TYR:H->  172:ASN:H
172:TYR:CA-> 172:ASN:CA
172:TYR:HA-> 172:ASN:HA
172:TYR:CB-> 172:ASN:CB
172:TYR:2HB->172:ASN:2HB
172:TYR:3HB->172:ASN:3HB
172:TYR:CG-> 172:ASN:CG
```

```
172:TYR:CD1->172:ASN:OD1
172:TYR:CD2->172:ASN:ND2
172:TYR:C->  172:ASN:C
172:TYR:O->  172:ASN:O
194:TYR:N->  194:ASN:N
194:TYR:H->  194:ASN:H
194:TYR:CA-> 194:ASN:CA
194:TYR:HA-> 194:ASN:HA
194:TYR:CB-> 194:ASN:CB
194:TYR:2HB->194:ASN:2HB
194:TYR:3HB->194:ASN:3HB
194:TYR:CG-> 194:ASN:CG
194:TYR:CD1->194:ASN:OD1
194:TYR:CD2->194:ASN:ND2
194:TYR:C->  194:ASN:C
194:TYR:O->  194:ASN:O
214:ASN:N->  214:SER:N
214:ASN:H->  214:SER:H
214:ASN:CA-> 214:SER:CA
214:ASN:HA-> 214:SER:HA
214:ASN:CB-> 214:SER:CB
214:ASN:2HB->214:SER:2HB
214:ASN:3HB->214:SER:3HB
214:ASN:CG-> 214:SER:OG
214:ASN:C->  214:SER:C
214:ASN:O->  214:SER:O
```

<u>mutant N294S to core</u>

```
172:TYR:C->     172:ASN:C
172:TYR:O->     172:ASN:O
172:TYR:N->     172:ASN:N
172:TYR:H->     172:ASN:H
172:TYR:CA->    172:ASN:CA
172:TYR:HA->    172:ASN:HA
172:TYR:CB->    172:ASN:CB
172:TYR:2HB->   172:ASN:2HB
172:TYR:3HB->   172:ASN:3HB
172:TYR:CG->    172:ASN:CG
172:TYR:CD1->   172:ASN:OD1
172:TYR:CD2->   172:ASN:ND2
194:HIP:C->     194:ASN:C
194:HIP:O->     194:ASN:O
194:HIP:N->     194:ASN:N
194:HIP:H->     194:ASN:H
194:HIP:CA->    194:ASN:CA
194:HIP:HA->    194:ASN:HA
194:HIP:CB->    194:ASN:CB
194:HIP:2HB->   194:ASN:2HB
194:HIP:3HB->   194:ASN:3HB
194:HIP:CG->    194:ASN:CG
194:HIP:ND1->   194:ASN:OD1
194:HIP:CD2->   194:ASN:ND2
214:SER:C->     214:SER:C
214:SER:O->     214:SER:O
```

```
214:SER:N->     214:SER:N
214:SER:H->     214:SER:H
214:SER:CA->    214:SER:CA
214:SER:HA->    214:SER:HA
214:SER:CB->    214:SER:CB
214:SER:2HB->   214:SER:2HB
214:SER:3HB->   214:SER:3HB
214:SER:OG->    214:SER:OG
```

mutant Y252H to core

```
172:HID:C->     172:ASN:C
172:HID:O->     172:ASN:O
172:HID:N->     172:ASN:N
172:HID:H->     172:ASN:H
172:HID:CA->    172:ASN:CA
172:HID:HA->    172:ASN:HA
172:HID:CB->    172:ASN:CB
172:HID:2HB->   172:ASN:2HB
172:HID:3HB->   172:ASN:3HB
172:HID:CG->    172:ASN:CG
172:HID:ND1->   172:ASN:OD1
172:HID:CD2->   172:ASN:ND2
194:HIP:C->     194:ASN:C
194:HIP:O->     194:ASN:O
194:HIP:N->     194:ASN:N
194:HIP:H->     194:ASN:H
194:HIP:CA->    194:ASN:CA
194:HIP:HA->    194:ASN:HA
194:HIP:CB->    194:ASN:CB
194:HIP:2HB->   194:ASN:2HB
194:HIP:3HB->   194:ASN:3HB
194:HIP:CG->    194:ASN:CG
194:HIP:ND1->   194:ASN:OD1
194:HIP:CD2->   194:ASN:ND2
214:ASN:N->     214:SER:N
214:ASN:H->     214:SER:H
214:ASN:CA->    214:SER:CA
214:ASN:HA->    214:SER:HA
214:ASN:CB->    214:SER:CB
214:ASN:2HB->   214:SER:2HB
214:ASN:3HB->   214:SER:3HB
214:ASN:CG->    214:SER:OG
214:ASN:C->     214:SER:C
214:ASN:O->     214:SER:O
```

Oseltamivir to core

```
388:UNK:C1-> 388:UNK:C2
388:UNK:C2-> 388:UNK:C5
388:UNK:C3-> 388:UNK:C7
388:UNK:C4-> 388:UNK:C8
388:UNK:C5-> 388:UNK:C1
```

```
388:UNK:C6-> 388:UNK:C4
388:UNK:C7-> 388:UNK:C6
388:UNK:C8-> 388:UNK:C3
388:UNK:C9-> 388:UNK:C9
388:UNK:N1 -> 388:UNK:N10
388:UNK:O1 -> 388:UNK:O11
388:UNK:O3 -> 388:UNK:O13
388:UNK:O2 -> 388:UNK:O12
388:UNK:H21-> 388:UNK:H10
```

Zanamivir to core

```
388:UNK:C1-> 388:UNK:C2
388:UNK:C2-> 388:UNK:C5
388:UNK:C3-> 388:UNK:C7
388:UNK:C4-> 388:UNK:C8
388:UNK:C6-> 388:UNK:C4
388:UNK:C7-> 388:UNK:C6
388:UNK:C8-> 388:UNK:C3
388:UNK:C9-> 388:UNK:C9
388:UNK:N15-> 388:UNK:N10
388:UNK:O17-> 388:UNK:O11
388:UNK:O18-> 388:UNK:O12
388:UNK:O19-> 388:UNK:O13
388:UNK:O20-> 388:UNK:C1
388:UNK:H15-> 388:UNK:H10
```

Appendix 5: mapping files for the SRSM calculations

WT to core mapping for H274Y calculations

```
194:HIP:C->     194:ASN:C
194:HIP:O->     194:ASN:O
194:HIP:N->     194:ASN:N
194:HIP:H->     194:ASN:H
194:HIP:CA->    194:ASN:CA
194:HIP:HA->    194:ASN:HA
194:HIP:CB->    194:ASN:CB
194:HIP:2HB->   194:ASN:2HB
194:HIP:3HB->   194:ASN:3HB
194:HIP:CG->    194:ASN:CG
194:HIP:ND1->   194:ASN:OD1
194:HIP:CD2->   194:ASN:ND2
```

WT to core mapping for N294S calculations

```
214:ASN:C->     214:SER:C
214:ASN:O->     214:SER:O
214:ASN:N->     214:SER:N
214:ASN:H->     214:SER:H
214:ASN:CA->    214:SER:CA
214:ASN:HA->    214:SER:HA
214:ASN:CB->    214:SER:CB
214:ASN:2HB->   214:SER:2HB
214:ASN:3HB->   214:SER:3HB
214:ASN:CG->    214:SER:OG
```

WT to core mapping for Y252H calculations

```
172:TYR:C->     172:ASN:C
172:TYR:O->     172:ASN:O
172:TYR:N->     172:ASN:N
172:TYR:H->     172:ASN:H
172:TYR:CA->    172:ASN:CA
172:TYR:HA->    172:ASN:HA
172:TYR:CB->    172:ASN:CB
172:TYR:2HB->   172:ASN:2HB
172:TYR:3HB->   172:ASN:3HB
172:TYR:CG->    172:ASN:CG
172:TYR:CD1->   172:ASN:OD1
172:TYR:CD2->   172:ASN:ND2
```

mutant H274Y to core

```
194:TYR:N->     194:ASN:N
194:TYR:H->     194:ASN:H
194:TYR:CA->    194:ASN:CA
194:TYR:HA->    194:ASN:HA
194:TYR:CB->    194:ASN:CB
194:TYR:2HB->   194:ASN:2HB
```

```
194:TYR:3HB->   194:ASN:3HB
194:TYR:CG->    194:ASN:CG
194:TYR:CD1->   194:ASN:OD1
194:TYR:CD2->   194:ASN:ND2
194:TYR:C->     194:ASN:C
194:TYR:O->     194:ASN:O
```

mutant N294S to core

```
214:SER:C->     214:SER:C
214:SER:O->     214:SER:O
214:SER:N->     214:SER:N
214:SER:H->     214:SER:H
214:SER:CA->    214:SER:CA
214:SER:HA->    214:SER:HA
214:SER:CB->    214:SER:CB
214:SER:2HB->   214:SER:2HB
214:SER:3HB->   214:SER:3HB
214:SER:OG->    214:SER:OG
```

mutant Y252H to core

```
172:HID:C->     172:ASN:C
172:HID:O->     172:ASN:O
172:HID:N->     172:ASN:N
172:HID:H->     172:ASN:H
172:HID:CA->    172:ASN:CA
172:HID:HA->    172:ASN:HA
172:HID:CB->    172:ASN:CB
172:HID:2HB->   172:ASN:2HB
172:HID:3HB->   172:ASN:3HB
172:HID:CG->    172:ASN:CG
172:HID:ND1->   172:ASN:OD1
172:HID:CD2->   172:ASN:ND2
```
